# Hydrologic Landscape Regionalisation Using Deductive Classification and Random Forests

**DOI:** 10.1371/journal.pone.0112856

**Published:** 2014-11-14

**Authors:** Stuart C. Brown, Rebecca E. Lester, Vincent L. Versace, Jonathon Fawcett, Laurie Laurenson

**Affiliations:** 1 School of Life and Environmental Sciences, Deakin University, Warrnambool, Victoria, Australia; 2 Greater Green Triangle University, Department of Rural Health, Flinders University, Warrnambool, Victoria, Australia; 3 Centre for Environmental Management, Faculty of Science and Technology, Federation University Australia, Ballarat, Victoria, Australia; 4 Sinclair-Knight-Merz, Bendigo, Victoria, Australia; Arizona State University, United States of America

## Abstract

Landscape classification and hydrological regionalisation studies are being increasingly used in ecohydrology to aid in the management and research of aquatic resources. We present a methodology for classifying hydrologic landscapes based on spatial environmental variables by employing non-parametric statistics and hybrid image classification. Our approach differed from previous classifications which have required the use of an *a priori* spatial unit (e.g. a catchment) which necessarily results in the loss of variability that is known to exist within those units. The use of a simple statistical approach to identify an appropriate number of classes eliminated the need for large amounts of *post-hoc* testing with different number of groups, or the selection and justification of an arbitrary number. Using statistical clustering, we identified 23 distinct groups within our training dataset. The use of a hybrid classification employing random forests extended this statistical clustering to an area of approximately 228,000 km^2^ of south-eastern Australia without the need to rely on catchments, landscape units or stream sections. This extension resulted in a highly accurate regionalisation at both 30-m and 2.5-km resolution, and a less-accurate 10-km classification that would be more appropriate for use at a continental scale. A smaller case study, of an area covering 27,000 km^2^, demonstrated that the method preserved the intra- and inter-catchment variability that is known to exist in local hydrology, based on previous research. Preliminary analysis linking the regionalisation to streamflow indices is promising suggesting that the method could be used to predict streamflow behaviour in ungauged catchments. Our work therefore simplifies current classification frameworks that are becoming more popular in ecohydrology, while better retaining small-scale variability in hydrology, thus enabling future attempts to explain and visualise broad-scale hydrologic trends at the scale of catchments and continents.

## Introduction

### Flow variability and ecological controls

Long-term trends in flow variability in streams have the ability to create and maintain ecosystem dynamics for a range of ecologically-important conditions [1], [2] and can therefore influence biotic communities and abiotic conditions [1] at local to regional scales, both temporally and spatially [1], [3]. These long-term trends are controlled by the same factors influencing the hydrologic cycle in a landscape and ultimately influence physical habitat and refuge availability, food distribution and abundance, and opportunities for migration, reproduction and recruitment [4]. Given this ability for hydrologic variability to control the ecological and biophysical attributes of in-stream and riparian systems, landscapes that have similar hydrologic properties should have similar biological and ecological assemblages [5]. Furthermore, if the same or similar hydrologic landscapes can exist in multiple spatial locations within bioregions, it stands to reason that the ecology of these systems should also be similar, regardless of spatial location. The ability to identify, classify, and validate spatial patterns in hydrologic landscapes is an important step in creating a solid foundation to assess the impact of natural flow variability, associated ecological conditions and management of water resources across a range of spatial scales. As such, hydrologic classification has been identified as a critical step in providing a spatially-explicit understanding of the magnitude and timing of flow regime variation within and between rivers and regions [2], [6].

### Landscape and hydrologic units

Landscape characteristics affecting the quality, quantity, and movement of water are extremely complex [7]. The earth is made up of a number of different landforms, geological settings and climatic conditions, and the idea of a simple, unifying conceptual hydrologic framework may seem impossible to achieve [7]. However, landscapes that appear unique and diverse often actually have a common set of attributes (e.g. governing the movement of water). Winter [7] introduced the concept of hydrologic landscape units, which suggests that the complete hydrologic system (i.e. incorporating surface runoff, groundwater flow and atmospheric water) interacts with simple physiographic features, and that these features then become the building blocks of all hydrologic landscapes. Therefore, by this rationale, the movement, storage and release of surface and subsurface water are controlled by a common set of physical principles regardless of the geographic location of the landscape [8]. Winter [7] termed these ‘fundamental hydrologic landscape units’ (FHLU), and defined the conceptual unit as a land surface form which includes an upland, an adjacent lowland and the valley side that separates them. The hydrologic system of an FHLU consists of: 1) the movement of surface water (controlled by the slopes and permeability of the landscape); 2) the movement, storage and release of groundwater (a function of the geologic setting); and 3) atmospheric water exchange (controlled by climate) [7]. Much peer-reviewed research supports the idea that all hydrologic landscapes can be considered to be variations and multiples of FHLUs, and that these can then be used to describe major, spatially-contiguous and discrete landscape types that should have similar hydrologic conditions (e.g. [6], [8], [9]). Since the concept was first introduced, further research has been conducted to delineate hydrologic landscape regions based on a number of different approaches and across a variety of scales (see Olden et al. [9] and Kennard et al. [6] for an extensive list of examples).

### Deductive and inductive landscape classification

Classification is the process of systematically placing objects into classes that are similar with respect to a set of variables or characteristics. Hydrologic classification is therefore the process of systematically arranging streams, rivers or catchments into classes that are similar with respect to their flow regime [6], [9]. While hydrologic classification can refer to a broad assortment of methods, a review by Olden et al. [9] recognises two broad approaches to hydrologic classification; deductive and inductive approaches (not to be confused with top-down and bottom-up logic; see below). The inductive approach uses the emergent properties of discharge time series data to generate classes. In contrast, the deductive approach to classification is used when attempting to describe broad spatial patterns in flow regime variability where there is a lack of gauged or modelled streamflow data available. Deductive methods of environmental classification are commonly used when the objective is to quantify and describe spatial variation in flow regime attributes. This approach to classification identifies groups on the basis of physical and climatic attributes that, over broad scales, produce similar hydrologic responses in stream systems [9]. The increased availability of high-quality, hydrologically-relevant spatial datasets (e.g. climate, topography, land use) makes deductive reasoning an appealing method when attempting to define spatial similarities or dissimilarities in hydrological characteristics [9]. It has been demonstrated that the deductive approach to hydrological classification can help in the prediction of streamflow metrics [10], [11], and that it improves predictive streamflow models when those models are stratified by hydrologic regions [12]. However, some facets of flow regimes (e.g. low flow magnitude and duration) are difficult to accurately characterise and quantify with this approach due to limitations in data quality and conceptual knowledge of the systems, and spatial variability of hydrological processes in many regions [6], [9], [13].

Wolock et al. [8] used the concept of hydrologic landscapes introduced by Winter [7] to classify nearly 44,000 catchments (∼200 km^2^ in area each) using a combination of multivariate ordination and cluster analyses. Kennard et al. [6] presented a method combining non-hierarchical clustering of climate, topography, soils and geology, vegetation and flow data to group Australian streams at a continental scale with mixed success. Sawicz et al. [14] employed the use of precipitation-temperature-streamflow signatures and Bayesian clustering to characterise 280 non-contiguous catchments located in eastern USA so as to understand similarities in climatic and landscape attributes across the region. Their work found that signatures which vary along climatic gradients exerted a stronger influence on cluster separation than those signatures which may vary as a result of geology or land cover. It has also been shown by McManamay et al. [13] that hydrological regionalisations [15] can be severely lacking in their ability to explain variation in a number of streamflow metrics.

The approaches by Wolock et al. [8], Kennard et al. [6], Sawicz et al. [14] and others all require the use of catchments or some choice of arbitrary spatial unit (e.g. eco/bio-region) to delineate and display the results of the clustering. However, there is evidence of significant flow variability within river catchments [6], [16] and significant spatial variability in climate and land use within sub-catchments that affect wetland extent [17]. The approach of delineating spatial units *a priori* leads to a loss of spatial variability, particularly as the catchment or spatial units become larger. Olden et al. [9] state that while deductive classification is common in the literature, hydrologic landscape regions and other similar concepts that are founded on physical principles have rarely been tested with this approach. The *a priori* (or ‘top-down’) specification of boundaries between classes has been criticised, while alternative ‘bottom-up’ approaches, where groups are developed as an emergent property of the data [18] (not to be confused with inductive reasoning which relies on time series hydrologic data) have been considered to be in keeping with physical ecohydrological principles [9]. Using a bottom-up approach, spatial and group clustering patterns are generated based on the analysis of a large number of units, such as pixels or micro-catchments. These units are then allocated into clusters based on their multivariate similarity [18]. However, a number of subjective choices as to which datasets to include, classification strategies and the number of groups in the classification process still need to be made. Such decisions could affect the quality and repeatability of the classification process when applied to different regions and datasets [9], [14], [18], [19]. Despite the potential limitations, the routine availability of these datasets and the application of statistical clustering and analyses have allowed scientists to begin to link spatial patterns to ecohydrological processes.

### Statistical clustering and multivariate analyses

Statistical clustering and multivariate analyses are important and powerful tools in the identification of spatial and temporal gradients. There is a multitude of variations on the theme of statistical clustering [20]–[22], but the most commonly used are hierarchical agglomerative methods [23] which fuse individual samples into like groups, gradually increasing the similarity within groups while lowering the similarity level between groups; i.e. each sample starts as its own group and pairs of groups are merged moving up a hierarchy. The process is considered complete when all samples are contained within a single group or cluster. Unlike hierarchical clustering, non-hierarchical clustering places samples into groups that are not related hierarchically, but differ from each other significantly in multivariate space. Described simply, non-hierarchical clustering tends to work by assigning each sample (*n*) into a pre-defined number of clusters (*k*) and then cluster membership of the samples is iteratively reassessed, usually with the criterion of maximising between-cluster variance while simultaneously minimising within-cluster variance. The most common example of non-hierarchical clustering is the *k*-means algorithm [24]. In some instances, the groups extracted by hierarchical and non-hierarchical algorithms do not differ significantly [25], but non-hierarchical methods can be much more efficient at extracting groups from large datasets [26].

Once statistical clustering has occurred, an analysis of the performance of the clustering can be conducted through the use of multivariate statistics and, in particular, ordination plots. There are a number of ordination techniques (e.g. principal components analysis (PCA), and multidimensional scaling (MDS)) that, broadly speaking, reduce multidimensional space so that objects can be compared graphically in two or three dimensions, without a significant loss of explanatory information. It is also possible to use the clustering information to train predictive models to classify samples not included in the original classification. This is where supervised classification algorithms, such as random forests (RF) [27], [28], coupled with geographic information systems (GIS) and image processing software can be applied to extend the applicability of deductive landscape classification approaches to regionalisation studies.

### Supervised classification of landscapes

One of the most common applications of remotely-sensed images and data is the creation of maps of vegetation type, soil properties or other discrete classes. In supervised classification, the location of known classes on those maps (i.e. training sites) is used by the software to determine the spectral signature of the pixels belonging to each of those classes. Each pixel in the image (i.e. outside the training sites) is then assigned, based on its spectral signature, to the class it most closely matches. Supervised classification can be applied at the individual pixel level or to groups of adjacent, similar pixels for the creation of contiguous regions. However, for the classification to work effectively, *a priori* knowledge of where the classes of interest (e.g. land cover types) are located is required. When supervised classification is combined with, for example, an unsupervised statistical classification, the process is referred to as hybrid (or semi-supervised) classification [29]. A major benefit of hybrid classifications for landscape regionalisation is that they permit the bottom-up approach to deductive classification as recommended by Mackey et al. [18] and Olden et al. [9]. This eliminates the need for survey approaches to develop *a priori* knowledge of the location of classes of interest which require expert opinion and substantial amounts of qualitative evidence which is not always available or suitable. The hybrid approach also eliminates the need to define a spatial unit a priori (e.g. a catchment) and allows small-scale (e.g. intra-catchment) variability to be identified and preserved where it may otherwise be lost.

### Aim of study

The aim of this study was to create a hydrologic landscape regionalisation using deductive reasoning and a bottom-up approach to statistical clustering combined with a hybrid classification. The regionalisation was then assessed based on its ability to discriminate between groups (regions) based on a number of streamflow indices. In this research, we used unsupervised classification (i.e. the statistical clustering) to first determine class membership based on multivariate space and then used supervised image classification to classify the remaining pixels from a number of ecohydrologically-important layers into the classes of interest as defined by the statistical clustering. This approach will permit the regionalisation of spatially non-contiguous regions, while maintaining small-scale intra-catchment variability that would be lost using catchments as the unit of classification as has often been done in the past. The assessment of the ability of the regions to differentiate among streams based on a number of flow indices provides insight into the utility of the method in predicting streamflow characteristics in ungauged catchments.

## Materials and Methods

**Figure 1:**
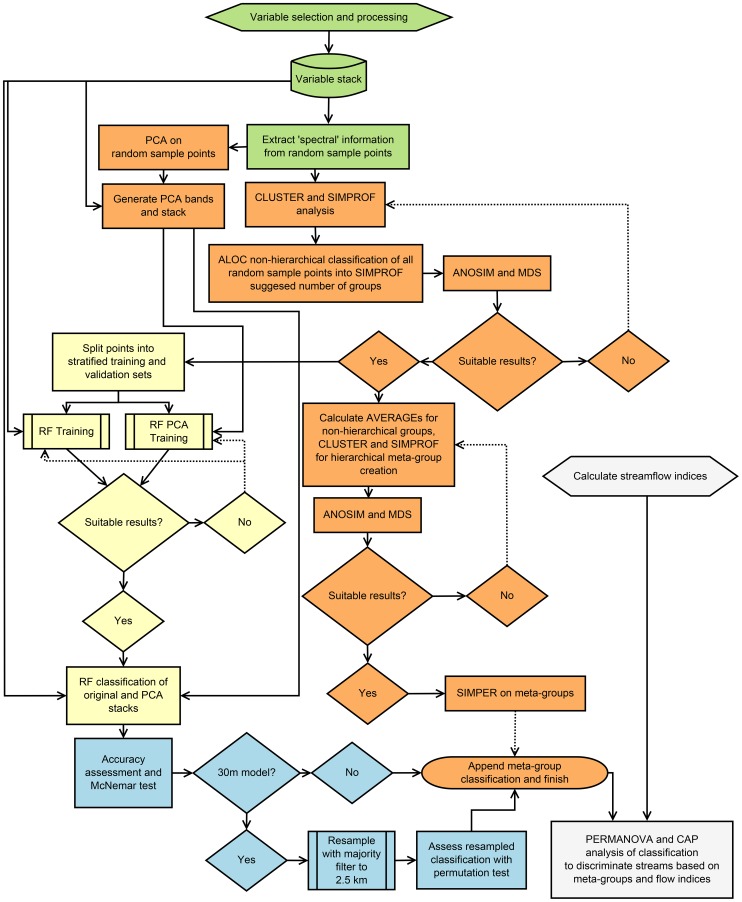
Graphical overview of the methods employed in the creation of the hydrological regionalisation; the training and validation of the RF models used to extend the statistical clustering to the state of Victoria; and of the validation of the classification with hydrological data. Each colour coded section of the figure corresponds to a section in the methods. Green: Variable Selection and Processing; Orange: Development of Classification Groups; Beige: Hybrid Classification with Random Forests; Light Blue: Accuracy Assessment; Grey: Relationship between the Regionalisation and Hydrologic Indices. The process for the ALOC 20 100% models did not involve splitting the ALOC classified random sample points into training and validation subsets and model accuracy was only assessed with OOB accuracy from EnMap-Box.

To clarify the process used in the creation of the hydrological regionalisation and the validation and training methods for the RF models, a graphical overview of the methods is presented in Figure 1.

### Site description

**Figure 2:**
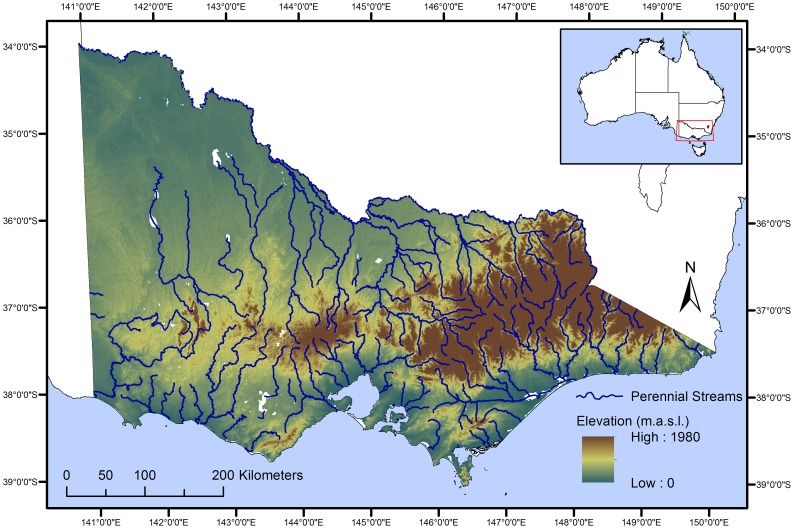
Location of the study area in south-eastern Australia. Dark blue lines represent perennial rivers, while the colour gradient represents elevations, with darker browns indicating higher elevations.

Victoria is the southernmost state of mainland Australia, comprising an area of 227,594 km^2^, and bordered by the southern bank of the Murray River to the north, South Australia to the west and separated from Tasmania by Bass Strait to the south. Topographically, geologically, and climatically, Victoria is diverse, varying from wet temperate climates in the southeast to alpine areas rising to ∼2000 m altitude in the northeast (Figure 2). To the west and northwest are extensive, flat areas of semi-arid plains, while most of the rest of the state experiences a Mediterranean climate consisting of hot, dry summer and cool, wet winters [30]. Median annual rainfall in Victoria exceeds 2,500 mm in some parts of the mountainous northeast but is less than 300 mm in a large part of the west and northwest [31]. Generally, snowfall is only observed in the mountains and hills to the east and centre of the state. Victoria has an extensive wetland system, with nearly 17,000 wetlands larger than 0.01 km^2^ in surface area [32], and a large river network, with the largest being the Murray River system.

### Site description of case-study area in western Victoria

The Glenelg-Hopkins region of western Victoria covers approximately 27,000 km^2^ and the regional cities of Warrnambool, Ararat, Hamilton, Portland and the western fringes of Ballarat are within its boundary. The region contains the Grampians Ranges in the north but is generally a low-lying series of catchments across three major catchments – Glenelg, Hopkins and Portland. The region has been previously studied with respect to land-use and land-cover changes [33] and the associated impacts on nutrient exports, in-stream salinity and dryland salinity [34], [35], while recent work has examined the spatio-temporal variability between land cover, climate and wetland extent [17], the impact of land-cover changes on groundwater levels [36] and empirically modelled streamflow response to land-use change [37].

### Variable selection and Processing

The first phase of the classification involved selecting suitable variables upon which to base our classification. Steps associated with variable selection and processing, described in this section, are outlined in green in Figure 1. Based on the concept of FHLUs, 25 variables were chosen that could explain the storage, movement, and quality of surface water, groundwater, and atmospheric water. A full list and brief description of each of the variables are presented in Table S1. All raster calculations and raster analysis for the processing of variables was conducted in ArcGIS 10.1 [38].

**Figure 3:**
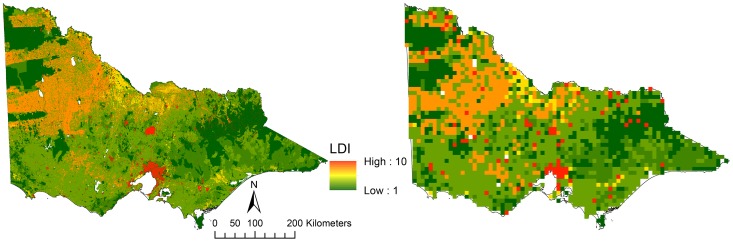
An example of the differences in resolution identified when working in relatively small study areas. The left hand image is the Landscape Development Intensity index (LDI) at a 30-m resolution while the image on the right is the LDI at a 10-km resolution. The accuracy of supervised classifications can be affected by the spatial resolution of the input images and as such we developed models at both resolutions.

The raster datasets employed in the study covered a wide range of resolutions (30 m–10 km). Typically, with GIS, analyses are only considered to be suitable if all rasters are resampled to the coarsest resolution. However, this can result in the loss of a substantial amount of detail and information and can affect the ability of supervised classification methods to successfully classify pixels (See Figure 3 for a comparison between the 30-m and 10-km Landscape Development Index (LDI)). Therefore, in this study, two approaches were used to standardise the scale of our raster data. The first approach was to resample all datasets to the finest resolution (30 m); and the second involved re-sampling all the raster datasets to the coarsest resolution found in our datasets (10 km). All rasters were continuous in their spatial coverage with the exception of the soil hydrological properties (KSAT, PAWC and soil horizon thickness) which had significant gaps where large lakes and wetlands were found. There was also a significant gap in coverage on the eastern headland of Port Phillip Bay. To ensure that all datasets aligned correctly and had the same degree of spatial continuity, the digital elevation model (DEM) was used as a snap raster for the resampling. Once the resampling had been completed using a nearest neighbour algorithm, the now 30-m soil properties were used as a mask to extract all other raster values. The result of this was that all of the datasets used in the analysis had a 30-m spatial resolution and all had corresponding areas of missing data that would be excluded from any analysis.

For the second approach, all of the original datasets were resampled to 10 km using a nearest neighbour algorithm and the mean annual evapotranspiration raster as the snap raster. Two different datasets were used as snap rasters so that the pixels of the resampled rasters (at either 30 m or 10 km) would be aligned correctly at the respective resolution. If this study had been conducted at a continental scale, then a coarser resolution would be more suitable, however as it was conducted on a relatively small scale, we considered that resampling to a finer resolution was both suitable and justifiable. Furthermore, limiting our analysis to a coarser resolution (on the basis of a single coarse-scaled dataset; mean annual evapotranspiration), would have significantly affected the applicability and usefulness of the method presented here, specifically, the ability of the RF model to accurately recover and reproduce allocated class information. Previous research has shown that the accuracy of supervised classifications, at both an overall and per class level, can be affected by the spatial resolution of the input images [39], [40]. As such, we assessed this issue through the accuracy of the RF model and the ability of the hybrid classifications to accurately recover the class information using the coarser dataset (see section on accuracy assessment). A layer stack of both sets of variables (30-m and 10-km resolution) was produced in ENVI 4.8 [41] for later use with the RF model.

Two random distributions of sampling points (*n* = 10,000 and *n* = 410; with minimum distances between points of 30 m and 10 km respectively) were then created. Ripley's *K* function [42], which determines whether features are significantly clustered or dispersed over a range of distances, was then used to assess whether both sets of points were distributed across our sampling area. Using the random-sampling points, raster values were extracted from each of the raster layers for later use in clustering, and then the training and validation of the RF model.

### Development of classification groups

The selected variables were then statistically analysed to develop the groups (known as ‘regions’) to be used as the basis for classification. The steps involved are outlined in orange in Figure 1. All sampling points that were found to contain missing data were removed prior to analysis, resulting in *n* = 9,958 (30-m data) and *n* = 406 (10-km data) for the two sets of data points. For the 30-m data, three 1000-point subsamples were taken for initial statistical analysis, while all data points were used for the 10-km dataset.

In order to develop a bottom-up approach to classification, where groups are an emergent factor of the data rather than defined *a priori*, we cluster-analysed each of the subsamples individually. Due to the large ranges and different scales used across our variable set, Gower similarity matrices were constructed for each of the initial analysis datasets using PRIMER 6 [43]. The CLUSTER [44] function was then used with a SIMPROF [45] test, to identify the number of statistically-significant (*α* = 0.05) groups within the datasets. Essentially, SIMPROF determines the number of significant groups with the assumption of no *a priori* groups by calculating similarities between every pair of samples using the chosen resemblance matrix and a hierarchical cluster dendrogram. Beginning at the top of an already-defined hierarchy (i.e. by the CLUSTER function), progression down the divisions or branches of the dendrogram is only permitted if the current set of samples is deemed to still have statistically significant dissimilarity. Upon encountering a non-significant result (i.e. the samples are similar), no further tests are performed down that branch of the dendrogram and all samples below are considered part of the same group [45]. A limitation of the SIMPROF test is that groups identified by the test may be at too fine a level of detail for practical purposes. However, if the resulting clusters are super-sets of the SIMPROF-defined groups, it is appropriate to define coarser groupings based on an arbitrary slice at some chosen level of similarity [45]. As the 30-m SIMPROF tests were conducted on three 1000-point subsamples, the subsample that produced the largest number of groups was used to determine the number of groups for the 30-m data. Even though SIMPROF uses a hierarchical relationship between sampling points to determine the number of clusters present in the data, we believe this approach is suitable for estimating an appropriate number of non-hierarchical groups as opposed to choosing an arbitrary *k* number of groups.

We then classified the full datasets (*n* = 9,958 and *n* = 406) into the number of classes suggested by the SIMPROF tests using the non-hierarchical clustering algorithm ALOC [26] and the Gower metric in PATN v3.1.2 [46]. Group allocations were then exported from PATN and joined to the original datasets as factors for further analysis. Once group membership information was in PRIMER, the ANalysis Of SIMilarities (ANOSIM; [44]) routine was used to test for statistically-significant differences among sample groups. Based on the *R* statistic, which is scaled to be between −1 and +1, global *R* values >0 indicate greater dissimilarity between groups than within groups [23]. Group averages were calculated using the AVERAGE [43] tool in PRIMER to visually analyse group separation using MDS. MDS is useful in providing a visual representation of the pattern of similarities between objects or groups while reducing the multidimensional space to be more readily interpretable (i.e. reducing data to two or three dimensions). The ability of MDS to reduce the degree of multidimensional space is measured with a stress value. Essentially, stress is the mismatch between distances between all samples in the plot in multidimensional space and the calculated estimate of their respective locations in two or three dimensions, with lower values indicating better representation [23]. The CLUSTER and SIMPROF routines were then used to hierarchically cluster the ALOC generated group averages into ‘meta-groups’. By definition, non-hierarchical groups are not linked based on their hierarchical multivariate relationship to each other, but rather are defined by their multivariate dissimilarity. As such, group *x* may not be closely related to group *y* but could be more closely related to group *z*. By hierarchically clustering our ALOC generated groups, were we able to determine which ALOC groups were more closely related to each other based on their multivariate means. A standardised Euclidean distance similarity matrix was then created and the SIMilarity PERcentage (SIMPER; [44]) routine was then used to analyse variable contribution to each of the meta-groups and to examine between meta-group similarity, while the Kruskal-Wallis [47] statistic was used to assess the ability of each of the variables to differentiate between clusters.

Previous studies (e.g. Wolock et al. [8]) have employed PCA [44] to reduce dimensionality and reduce multi-collinearity among variables. Our method relied firstly on using ‘raw’ data (i.e. the data was not transformed in any way) to extract spectral information for the statistical clustering and then classification. The results of this method were then compared against a classification based on PCA-transformed data. PCA is a procedure where possibly correlated variables are orthogonally transformed into a new set of linear, uncorrelated variables known as principal components [23]. The transformation results in the first component explaining most of the data variance (i.e. the first component explains as much of the multivariate data as possible) while each additional component in turn is then created to explain the remaining variance. The number of components is less than or equal to the number of original variables and each additional component is created under the condition that it is uncorrelated with all of the preceding components [23]. Here, a PCA was conducted in PRIMER on the same standardised Euclidean distance matrix used for the SIMPER analysis. The eigenvectors, for the first five principal components (eigenvalues ≥1) were used in ArcMap to generate PCA bands and new PCA raster stacks were created in ENVI.

### Hybrid classification with Random Forests

The regions that were developed in the previous step were then used to classify the raster stack of the variables for the entire study area. Steps in this section are outlined in beige in Figure 1. RF is an ensemble machine-learning method used in classification and regression [27]. The RF method is relatively unknown in land remote sensing and has not been thoroughly evaluated by the remote sensing community, although it has been shown to be more accurate than single decision-tree classifiers [48]. RF requires two parameters for generating a predictive model: the number of trees (*k*) and the number of variables used for growing the trees (*m*). Therefore, a dataset can be classified by defining a constant number of *m* variables, while each of the training samples is classified by *k* trees. Classification is determined by using the mode of the classes output by individual trees for each training site 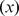, 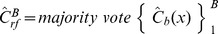, where 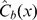 is the class prediction of the *b*th RF tree from a possible 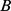 classes [48]. RF increases the diversity of the constituent trees by making them grow from different training data through bootstrap aggregation which involves random re-sampling (without deletion) of the original training dataset [27]. Therefore some data may be used more than once in the training of the model, while some may not be used at all [48]. Being an ensemble method, multiple models (trees) are used allowing the algorithm to obtain better predictive performance than that which would be obtained by using any of the constituent models individually. RF is becoming increasingly popular in data mining, remote sensing and landscape ecology as it is non-parametric, can generate internal, unbiased error estimates and variable importance, is robust to training data reduction and noise, and is highly accurate [27], [48].

In order to develop the RF models, the sampling points were randomly split into independent training (80%) and validation (20%) datasets and then stratified using the cluster-membership allocations from PATN. Due to the small sample size of the 10-km dataset, an additional RF model was created using 100% of the sample for RF training. Using ENVI, layer stacks of the raster data (30 m, 10 km, and PCA at both resolutions) were constructed and masked. Layer stacks, masks and training regions of interest were imported in to EN-Map Box 1.4 [28] to permit the building of RF models and the classification of the image stacks. EN-Map Box was set to use 200 trees (*k*) per sample, and the square root of the number of input variables on the non-PCA transformed data (*m* =  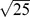  = 5), or *m-1* variables (*m* = 4) for the PCA models. The Gini coefficient [49] was used to calculate impurity, which is one method used to evaluate the best split decision for each tree.

### Accuracy assessment

The ability of the classification to accurately represent the information across a range of resolutions was then tested, with the relevant steps outlined in light blue in Figure 1. The accuracy of the RF models in recovering and classifying the image stacks into the ALOC classes was assessed with out-of-bag (OOB) error rates generated in EN-MAP box, and using the independent 20% validation dataset to calculate percent agreement between classified and validation data, user and producer accuracies, and Kappa (κ) coefficients [50] in ENVI and R 3.0.1 [47] with the *psych* [51] and *irr* packages [52]. User's accuracy refers to the probability that a pixel classified into a certain class really belongs to that class, while producer's accuracy refers to the probability that a certain class is classified correctly. The locations of the validation pixels were used to extract class information from the original and PCA classifications and percent agreement and κ coefficients between model types were examined. High levels of agreement between the original and PCA classifications would indicate an insignificant amount of multivariate information loss by PCA and further support the use and application of methods that reduce data dimensionality and multi-collinearity between variables in regionalisation studies. The 10-km sample that used 100% of the data for RF training could not be assessed for accuracy independently and was therefore only assessed with OOB error and class distributions.

We also decided that, due to the resampling of the original data (to 30-m from a range of resolutions), it was worth investigating the effect of resampling the 30-m classifications to a coarser resolution (using the majority filter) to help remove some of the finer-scale variability in the data. A resolution of 2.5 km was chosen as a suitable pixel resolution, to ensure that our resampled assessment points were further apart than the mean distance of the original samples (see below), this meant our resampled classifications were equal to or larger than the resolution of the majority of the datasets while still being finer than the soil erosivity index. To assess agreement between the 30-m and 2.5-km resampled classifications (i.e. original and PCA both at 30-m and resampled 2.5-km), 300 random validation points were selected from the 20% validation datasets, with minimum distances between points of 2.5 km, and used to extract class information from the 30-m and 2.5-km resampled classifications. Agreement between the validation data and the 30-m and 2.5-km resampled classifications was assessed with percent agreement and κ coefficients between model resolutions. Visual inspection of the class distributions was also conducted; for this the 300 random validation points were used for the 30-m and 2.5-km classifications, while all 81 validation points for the 10-km classifications were used. McNemar's chi-square test [53], [54] was used to formally test for statistically-significant differences between model types (e.g. 30-m original and 30-m PCA) and resolutions (e.g. 30-m original and resampled 2.5-km original) and the validation dataset using the same 300 random validation points; however we recognised *a priori* that the relatively large number of random validation points would likely make any difference statistically significant and that the resolution tests would not be independent of one another. As such, a permutation- based method (*n* = 9999) was also used to assess for statistically-significant differences in the κ values of the classifications [55], [56]. The algorithm worked as follows:

1. If V =  validation data; A =  classification 1; B =  classification 2
2. Let test(*x*, *y*), be a function that calculates the test statistic (κ) for the classifications,
3. H_0_  =  if A and B are approximately equal in their classification accuracy, observations in A and B can be exchanged without affecting κ, given by test(V, A) and test(V, B),
4. Randomly exchange data between A and B, *n* times, and observe how these changes affect the κ of A and B, i.e. test(V, A) – test(V, B),
5. *n* permutations would result in *N* data points. Rank the *N* data points and observe where the κ from the original test (i.e. not the permutated data) is located among the κ values from the permutated data points. If the original κ is outside the 0.975 percentile (or below the 0.025 percentile) then we can claim that the two classifications are different at α = 0.05.

If a high level of agreement was found, as indicated by high percent agreement and high κ, then resampling to 2.5-km *a posteriori* could be considered a suitable and justifiable method for smoothing the finer-scale variability.

### Relationships between the regionalisation and hydrologic indices

Finally, we tested the results of our classification against a traditional classification based on hydrologic indices. Steps in this section are outlined in grey in Figure 1. As a preliminary assessment of the ability of the regionalisation to differentiate among streams with differing hydrology, we calculated a range of streamflow indices based on the recommendations of Olden and Poff [57] and then explored the relationships between the regions and streams with a permutation-based ANOVA (PERMANOVA) [58] and a constrained discriminant ordination (Canonical Analysis of Principal Coordinates (CAP); [59], [60]).

Streamflow gauge locations were downloaded from the Water Measurement Information System (data.water.vic.gov.au/monitoring.htm). These locations were then overlayed on the regionalisation (30-m ALOC 23 meta-group classification, see results) and had group (region) information appended to them. In regions where there were more than 50 stream-gauges present, 50 were chosen at random to be included in the analysis. Daily streamflow data between 1980 and 2010 was then downloaded for 564 gauges throughout Victoria. A minimum record length of 15 years within the 30-year temporal window was required for a gauge to be included in the analysis [61]. Stream gauges that were potentially subject to modification by weirs, dams or water extractions were not specifically excluded from the analysis. Where there were missing periods of flow information (to a maximum of 20 days in any single event) the record was in-filled using linear interpolation [62] with the Time Series Manager module of the River Analysis Package (RAP) [63]. Gauges that had a single period of missing data greater than 20 days were excluded from the analysis. Thirty-two indices characterising different aspects of the flow regime (Table III, All Streams; [57]) for each stream were calculated using the Time Series Analysis module of RAP. Indices that were related to discharge (i.e. those divided by catchment area) were not included in the analysis.

To test for differences among our groups, the PERMANOVA+ add-on [64] for PRIMER was used. One-way PERMANOVAs, (999 permutations) using Group as a fixed factor, were run for the dataset of flow indices based on the original gauges (*n* = 201) and for an additional dataset consisting of a bootstrapped sample of those flow indices (*n* = 383), based on normalised Euclidean distance matrices. Analyses tested both for main effects and pairwise differences among groups. Traditional Analysis of Variance (ANOVA) is powerful for univariate data however the traditional multivariate analogues (e.g. MANOVA), are too stringent in their assumptions (in particular, that of multivariate normality which is frequently untrue in ecological data [25], [58]), for use in ecology. As such, permutation-based non-parametric methods are preferred [58]. PERMANOVA uses permutation methods to test the simultaneous response of one or more variables to one or more factors in an analysis of variance. The use of permutations in PERMANOVA removes the assumption of normal distributions which are required for traditional ANOVA/MANOVA testing and, as such, the only assumption of the test other than independence is that the observations can be exchanged under a true null hypothesis [58]. Another benefit of using a permutation approach is that the permutated *P*-values provide an exact test of each *individual* null hypothesis of interest, and as such ad-hoc pairwise corrections (e.g. Bonferroni) are not strictly necessary [64].

When data are classified into *a priori* groups, unconstrained ordinations (PCA, MDS) are extremely useful for visualising patterns from a multivariate space. However, the overall dispersion of points (when reduced to two or three dimensions) can often hide the true multivariate differences among those groups and it may be very possible to discriminate among groups through another direction or dimension of the multivariate data [64]. Unlike unconstrained ordinations, constrained ordinations have an *a priori* hypothesis which controls the way the multivariate data can be interpreted in an attempt to relate predictor variables (streamflows indices) to response variables (groups) [60]. In a discriminant analysis, the ordination axes are interpreted in such a way as to maximise the differences between *a priori* groups, while in a canonical correlation, the axes are interpreted to maximise correlations among variables. CAP first calculates the principal coordinate axes (PCO) among *N* samples, and then chooses an appropriate number of PCO axes (*m*) for interpretation based on a number of criteria (see Anderson et al. [64] for details), including a leave-one-out cross validation procedure which attempts to maximise classification success. The benefits of CAP over other constrained ordination methods are its ability to use any distance or dissimilarity measure, conduct permutation tests for significance of relationships among variables and predict group membership of new samples [60]. To assess the ability of our regionalisation to discriminate groups based on streamflow indices, three CAPs were conducted using the PERMANOVA+ add-on for PRIMER. Two of the analyses were performed against the group information extracted for each stream gauge using the same normalised Euclidean distance matrices that were used in the PERMANOVA tests (i.e. the original dataset and the bootstrapped dataset of hydrologic indices). The number of axes (*m*) was not specified and a permutation test (*n* = 999) was conducted to test the strength of the relationship. In addition, a third CAP was conducted using the bootstrap dataset where a stratified random sampling approach was used to remove 20% of the group information as a validation sample. The CAP was conducted as before, with the exception being that the validation cases were allocated groups based on the results of the CAP. Allocation accuracy by CAP was assessed by calculating percent agreement between the CAP allocated group and the original group information, and κ coefficients with the *irr* and *psych* packages in R. Pearson's r [47] was calculated between the between the number of gauges in each group and the number of gauges classified correctly from each group to assess for thresholds at which a specified level of accuracy could be achieved.

## Results

### Spatial distribution of sample points

Analysis of spatial distribution of the sampling points concluded that the mean, minimum and maximum distances to the nearest neighbour among the 30-m points were 2.4 km, 202 m and 8.8 km, respectively. The corresponding values for the 10-km points were 15 km, 10 km, and 47.4 km, respectively. Ripley's *K*, based on 999 permutations, indicated significant over-dispersion of the 30-m sample points to a distance of ∼250 m and a significant clustering at distances greater than ∼650 m, while the 10-km sample points were significantly over-dispersed at distances less than ∼12 km, but were significantly clustered at distances greater than ∼18 km. Based on these values, the spatial distribution of our sampling points was considered suitable for the analyses as no significant clustering was displayed by the sample points at the resolution of the datasets they would be sampling (e.g. there was no significant over-dispersion or clustering at the mean distance of 15 km for the 10-km points).

### Clustering and ordination

Twenty-three groups were identified within the 30-m data points and 20 groups for the 10-km data. As a result, the data were allocated into 23 (ALOC 23: 30-m data) and 20 (ALOC 20: 10-km data) non-hierarchical groups. There were wide variations in the ability of the model variables to differentiate among clusters (Table S2) and the group populations had differing multivariate distributions. The allocated groups were well separated (Figure S1) with global-*R* values of 0.852 (*p* = 0.001) for the ALOC 23 clustering and 0.908 (*p* = 0.001) for the ALOC 20 clustering, indicating that cluster membership was highly unlikely to be a result of chance alone. This was supported by the fact that neither ANOMSIM resulted in any permutations that had *R*-statistics greater than or equal to the global-*R* value.

The allocated groups were then further clustered into hierarchical meta-groups. Using the group averages for ALOC 23 and ALOC 20, 11 and ten meta-groups were generated (Figure S1 and Figure S2). ANOSIM analysis again indicated that cluster membership was highly unlikely to be a result of chance alone and suggested that the meta-groups for both the ALOC 23 and ALOC 20 models were well separated with global-*R* values of 0.668 and 0.762 (*p* = 0.001). Again no permutations had *R*-statistics that were greater than or equal to the global-*R* value. Some variables exhibited no relationship between the observed values and the meta-groups, while other variables show very clear relationships to the meta-groups (Figures S3–S7). For example, the values for the aridity index (low aridity index values represent drier regions) and rainfall decreased from groups A to K. The opposite relationship was observed for maximum and minimum temperatures, again suggesting as we move through regions A to K, the environment becomes drier and warmer. The BioClim variables 8, 9, 15 and 16 also supported this relationship, with increases in BIO08 and BIO09 (mean temperature of the wettest and driest quarter, respectively), and decreases in BIO16 and BIO17 (precipitation of the wettest and driest quarters, respectively). Variable percentage contributions to the meta-groups for both the ALOC 23 and ALOC 20 classifications differed markedly (Table S2, Figure S8) with, for example, saturated hydraulic conductivity of the B soil horizon (B_KSAT) contributing 0% to a number of ALOC 23 meta-groups, while contributing 46% to ALOC 23 meta-group F. The within-group variation was highly variable among groups, with average squared distances of groups ranging from 4.18 to 11.77 for the ALOC 23 meta-groups, and 4.44 to 13.45 for the ALOC 20 meta-groups (Table S2).

Ordination by PCA resulted in the creation of five principal components (PC) for both ALOC 23 and ALOC 20, using a minimum eigenvalue of one. The first PC for each of the ALOC 23 and ALOC 20 clustering explained 45% and 53% of the data variance, with eigenvalues of 11.2 and 13.2, respectively (Table 1). The five PCs combined explained 78% and 87% of the variance, with the final PC having eigenvalues of 1.39 and 1.25, respectively. PC 1 can be interpreted to represent those areas that are wet, cool, heavily vegetated, high elevation environments with steep slopes and low erodibility soils (Table 1, 0.2<*r*<−0.2). With the exception of PC 1, none of the PCs for either of the datasets can be interpreted to represent similar environments across the two classifications. For example, PC 2 of the ALOC 23 dataset can be interpreted to represent areas with thick, weathered, A-horizon soils with high levels of plant available water, with relatively small variations in temperature seasonality and poorly developed B-horizon soils. For the ALOC 20 dataset, on the other hand, PC 2 suggests areas of poorly developed A-horizon soils, but with thick B horizon soils, and cooler, wetter summers.

**Table 1:** Results of Principal Components Analysis.

ALOC 23						ALOC 20				
	PC1	PC2	PC3	PC4	PC5	PC1	PC2	PC3	PC4	PC5
*Eigenvalue*	11.2 (45)	3.26 (58)	2.07 (66.3)	1.58 (72.6)	1.39 (78.2)	13.2 (52.7)	4.04 (68.9)	1.68 (75.6)	1.58 (81.9)	1.25 (86.9)
*Variable*										
A_KSAT	0.14	0.14	**−0.42**	**−0.3**	0.18	0.13	**−0.22**	0.15	**0.53**	0.03
A_PAWC	0.01	**0.48**	−0.18	−0.02	−0.12	0.05	**−0.45**	0.12	0.1	−0.07
A_THICK	0.1	**0.45**	**−0.21**	−0.12	−0.02	−0.04	**−0.45**	0.1	−0.04	−0.12
ARIDITY_INDEX	**0.29**	0.01	0.04	0.02	0.03	**0.26**	−0.09	0	−0.03	−0.02
B_KSAT	0.15	0.05	**−0.4**	**−0.31**	0.2	0.16	**−0.22**	**0.26**	**0.29**	−0.08
B_PAWC	0.12	**−0.34**	−0.09	**−0.41**	**0.26**	0.17	0.18	0.01	**0.47**	**0.29**
B_THICK	0.06	**−0.38**	0.14	**−0.38**	0.16	0.1	0.3	−0.1	**0.35**	**0.42**
BIO04	−0.08	**−0.34**	**−0.29**	0.08	**−0.39**	**−0.21**	−0.01	**−0.26**	**0.26**	**−0.36**
BIO08	**−0.22**	0.05	−0.1	0.2	**0.44**	**−0.25**	−0.01	0.19	0.09	0.07
BIO09	**−0.24**	−0.1	−0.17	−0.04	−0.19	0.02	**−0.26**	**0.35**	**−0.26**	**0.52**
BIO15	0.08	0.14	0.16	**−0.43**	**−0.53**	**0.26**	−0.09	0	−0.11	0.1
BIO16	**0.28**	0.03	0.07	−0.05	−0.05	**0.26**	0.01	−0.19	0.03	−0.13
BIO17	**0.27**	−0.02	0.03	0.18	**0.23**	−0.04	**0.38**	**0.38**	−0.08	−0.01
ELEVATION	**0.25**	−0.14	−0.08	0.04	−0.16	**0.24**	0.18	0.11	−0.11	−0.01
ET_ANNUAL	**0.24**	0.14	**0.28**	0.06	0.1	**0.21**	−0.1	**−0.41**	−0.09	0.1
GW_SWL	0.17	−0.08	**−0.29**	0.09	−0.14	**0.23**	0.11	0.2	0.05	**−0.24**
GW_TDS	−0.17	−0.1	**−0.32**	0.08	0.02	−0.16	0.11	**0.41**	0.13	**−0.25**
LDI	−0.11	−0.11	0.16	−0.07	0.05	−0.15	−0.08	**−0.26**	0.18	0.08
MAX_TEMP	**−0.27**	−0.05	−0.18	0	−0.07	**0.26**	−0.09	0.01	−0.03	0
MIN_TEMP	**−0.25**	0.08	−0.05	0	0.15	**0.25**	0.02	0.07	0.08	**−0.21**
RAIN_ANNUAL	**0.29**	0	0.04	0.03	0.02	**0.27**	−0.02	0.04	0.01	−0.07
SLOPE_RAD	**0.23**	−0.1	−0.2	**0.22**	−0.03	**−0.26**	0.09	0.05	0.1	−0.04
SOIL_EROS	**0.26**	−0.07	−0.05	0.09	0.03	**−0.24**	−0.16	−0.07	0.13	0.01
TWI	−0.18	−0.01	0.16	**−0.27**	0.03	**−0.23**	0	0.03	−0.06	0.1
WEATH_IND	−0.16	**0.23**	0.1	**−0.26**	0.14	−0.19	−0.17	−0.03	0.11	**0.27**

Eigenvalues are presented in the top row while the numbers in brackets represents the cumulative % variance explained by the PCA. Bold numbers indicate variables with a correlation ≥0.2 or ≤−0.2 which was used for PC interpretation.

### Random Forests

#### Training and validation

**Figure 4:**
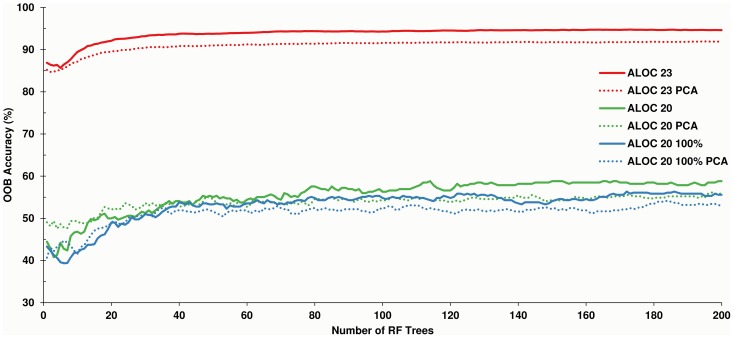
Out-Of-Bag (OOB) percent accuracies for the ALOC clusterings as classified by Random Forests. The 30-m ALOC 23 and ALOC 23 PCA models were significantly more accurate than the 10-km ALOC 20 classifications.

Two hundred trees were sufficient for the RF models to achieve acceptable accuracies. Exploratory analysis, using models with 5000 trees (not presented) showed little improvement in OOB error (<1%). OOB error rates were low for the ALOC 23 and ALOC 23 PCA RF models, with estimated maximum accuracies of 95% and 92%, respectively. The OOB error rate of the ALOC 20, ALOC 20 PCA and the 100% ALOC 20 and 100% ALOC 20 PCA models (the latter two were created due to the small sample size for the 10-km dataset as described above) was significantly worse, with estimated accuracies of 59%, 56%, 56% and 54%, respectively (Figure 4).

**Figure 5:**
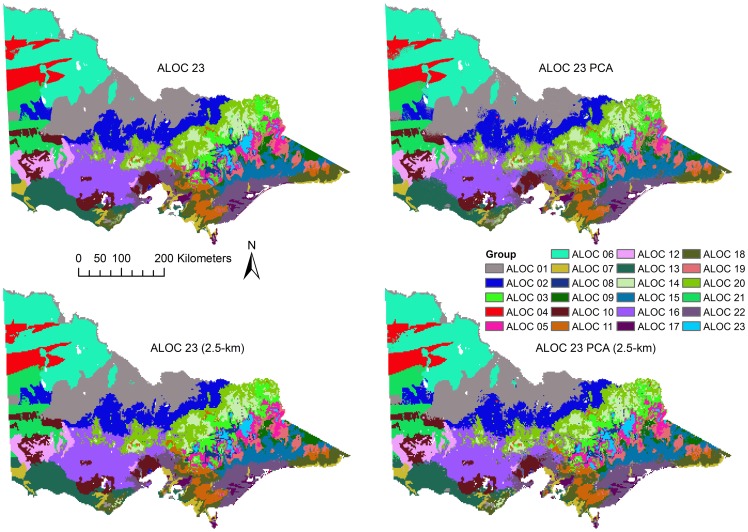
Results of the ALOC 23 (30 m) classifications. Top row - ALOC 23 and ALOC 23 PCA; Bottom row - ALOC 23 and ALOC 23 PCA resampled to 2.5 km. Colours represent each of the ALOC non-hierarchical clusters. Similar colours and cluster numbers do not necessarily represent related groups.

**Figure 6:**
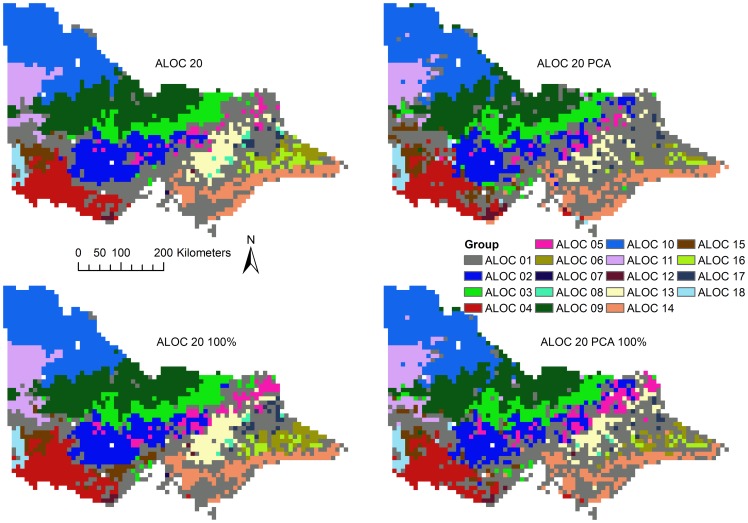
Results of the ALOC 20 (10 km) classifications. Top row - ALOC 20 and ALOC 20 PCA; Bottom row - ALOC 20 (100%) and ALOC 20 PCA (100%). Note that not all ALOC clusters are present in the final classifications. Colours represent each of the ALOC non-hierarchical clusters. Similar colours and cluster numbers do not necessarily represent related groups.

Classification accuracy was 95% (κ = 0.94) for the ALOC 23 classification and 92% (κ = 0.92) for the ALOC 23 PCA classification (Table S3) when tested against the validation dataset. The accuracy of the ALOC 20 and ALOC 20 PCA classifications decreased relative to those estimated by the RF OOB error, with accuracies of 46% (κ = 0.42) and 47% (κ = 0.44) (Table S3). The producer accuracies differed significantly for each of the classifications (Table S3), with observed minimum producer classification accuracies of 81% for the ALOC 23 classification and 59% for the ALOC 23 PCA classification. Likewise, the ALOC 20 and ALOC 20 PCA classification also exhibited low producer accuracies with minima of 0% observed for a number of classes in each classification. Visual inspection of the resulting classifications showed few obvious differences among the various ALOC 23 classifications (Figure 5), but more differences were apparent among the ALOC 20 classifications (Figure 6).

#### Comparisons between original, PCA and resampled classifications

**Figure 7:**
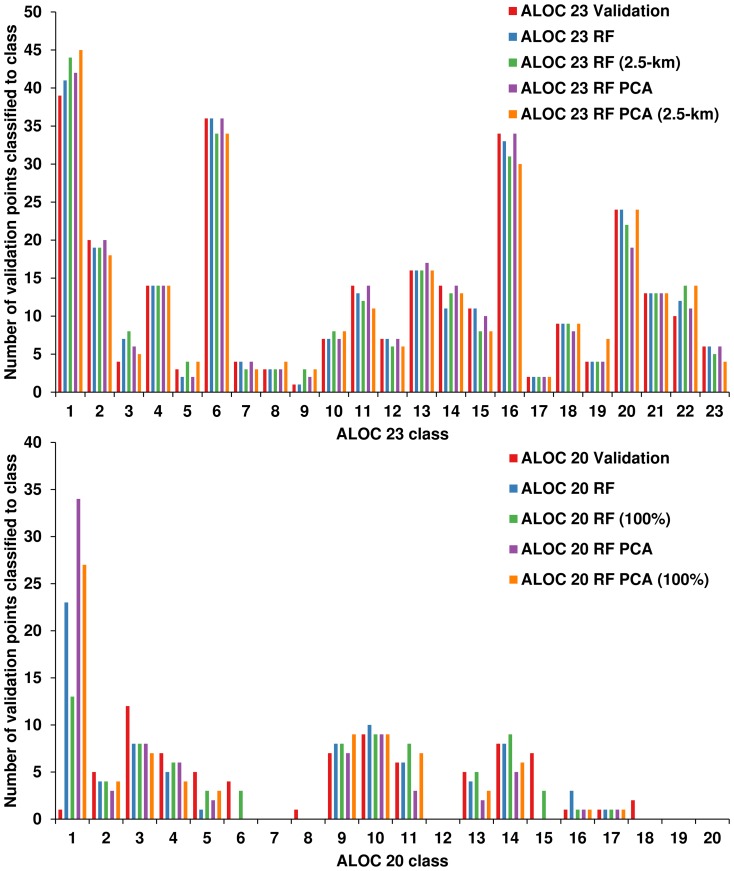
Class distributions from the final classified images. The 20% validation points were used to extract class information from the classified images. The resulting figure shows that the ALOC 23 classifications were much better at maintaining the class distribution of the validation dataset (shown above in red) than the ALOC 20 classifications.

The class distributions of the RF and resampled classifications illustrate that the RF classifications at 30-m and resampled to 2.5-km (bottom row, Figure 5) were quite successful in maintaining the distribution of the validation dataset (Figure 7). All 10-km models were missing classes 7, 8, 12, 18, 19 and 20 from the validation dataset (Figure 7), meaning that accuracy assessment of these classes was not possible, although classes 7, 8, 12, and 18 were present in the final classification (Figure 6). Class 1 tended to be over-classified by the ALOC 20 RF models, as evidenced by the large number of validation points classified as such (Figure 7). The ALOC 20 100% classification was also the only classification to have classes 6 and 15 represented at the validation locations.

Agreement between the ALOC 23 and ALOC 23 PCA classifications was high at 93% (κ = 0.93). There was no statistically-significant difference between the two classifications (χ^2^ = 2.72, *p* = 0.1). Agreements for the ALOC 20 and ALOC 20 PCA classifications was lower than that observed for the ALOC 23 classifications, at 72% (κ = 0.67), which was also non-significant (χ^2^ = 0.24, *p* = 0.63). This indicates that the classification based on the five principal components captured the vast majority of the variability among points at the 30-m scale, and most of the variability at the 10-km scale.

Visual inspection of the resampled classifications (bottom row, Figure 5) showed very similar results to the original 30-m classifications. Agreement between the resampled ALOC 23 classification and the original 30-m classification was high (92%, κ = 0.92) and an accuracy of 91% (κ = 0.9) was observed with the 300 randomly chosen points from the validation dataset. This was a 4% difference in accuracy to the original 30-m ALOC 23 classification. However, McNemar's test suggested the difference in classification accuracy was statistically significant (χ^2^ = 12.56, *p*<0.001). As the two classifications are not independent, the permutation test was also used to compare the two. This supported the results of the McNemar's test and a permutated κ difference of 0.062 was deemed significant (*p*<0.001). The ALOC 23 PCA resampling results were similar, with a relatively high agreement with the original 30-m PCA classification (88%, κ = 0.87), and 87% (κ = 0.86) agreement with the validation dataset, a 5% difference compared to the original ALOC 23 PCA classification. These differences were also statistically significant (McNemar's test, χ^2^ = 11.76, *p*<0.001; permutation test, κ difference  = 0.073, p<0.001). Agreement was quite high between the 2.5-km resampled ALOC 23 and 2.5-km resampled ALOC 23 PCA classifications (93%, κ = 0.92), however unlike the 30-m classification results, when comparing the agreement between the two resampled classifications McNemar's test suggested the results were significantly different (χ^2^ = 5.88, *p* = 0.015). As these two classifications were independent of one another the permutation test was not required.

**Figure 8:**
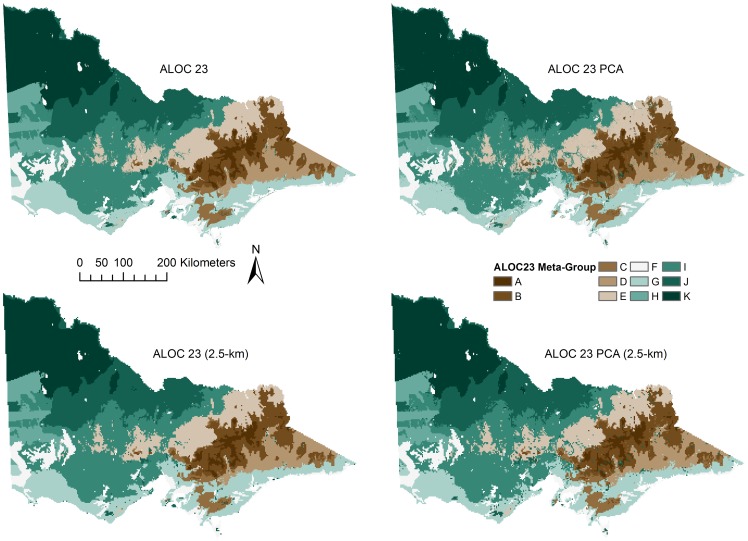
Results of the ALOC 23 (30 m) meta-group allocations. Top row - ALOC 23 and ALOC 23 PCA; Bottom row - ALOC 23 and ALOC 23 PCA resampled to 2.5 km. Colours represent each of the hierarchical meta-groups as defined by SIMPROF. Similar colours and group letters indicate a closer relationship than those further apart.

Once the classification assessment was finalised, the meta-group allocations were appended to the classifications. The ALOC 23 meta-group allocations were examined visually (Figure 8) and were deemed suitable given that regions having similar groups (e.g. A and B, represented by distinct colours in Figure 8) exist in similar areas among the two classifications and show quite obvious spatial relationships. While the performance of the ALOC 20 models was weaker, the meta-group assignment results were similar to those observed for the ALOC 23 classification in that similar meta-groups existed closer spatially (not presented).

### Spatial variability in western Victoria

**Figure 9:**
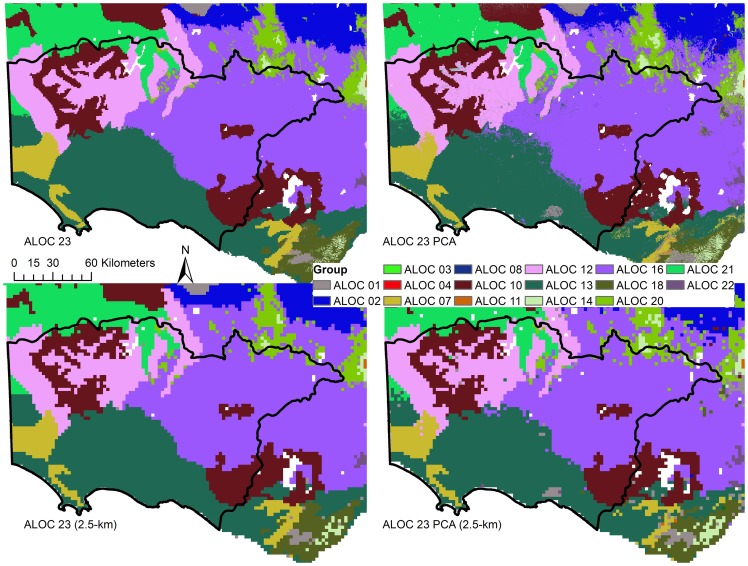
Results of the ALOC 23 (30 m) classifications for the Glenelg Hopkins region. Top row - ALOC 23 and ALOC 23 PCA; Bottom row - ALOC 23 and ALOC 23 PCA resampled to 2.5 km. Colours represent each of the ALOC non-hierarchical clusters. Similar colours and cluster numbers do not necessarily represent related groups. More spatial variability in the ALOC clusters is obvious in the Glenelg catchment (western and NW side), than that in the Hopkins (eastern) and Portland (south central) catchments.

The variability of model results was not uniform across the entire Glenelg-Hopkins case-study region (Figure 9). While the eastern and central parts of the region appeared to be relatively spatially uniform (i.e. they do not show a significant amount of variation in ALOC groupings); the western and north-western parts of the region are comprised of a number of ALOC classes. This suggests that the hydrological system varies spatially across the region, with the most variability likely occurring in the western half of the catchment.

**Figure 10:**
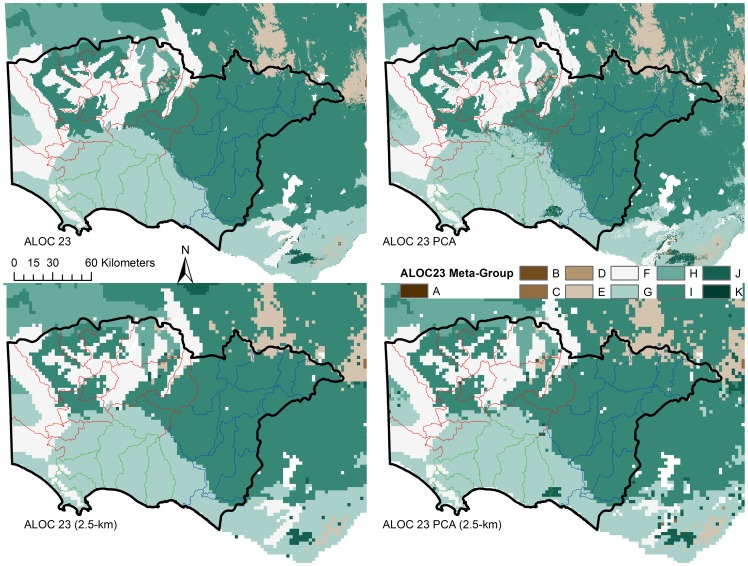
Results of the ALOC 23 (30 m) meta-group allocations for the Glenelg Hopkins region. Top row - ALOC 23 and ALOC 23 PCA; Bottom row - ALOC 23 and ALOC 23 PCA resampled to 2.5 km. Colours represent each of the hierarchical meta-groups as defined by SIMPROF. Similar colours and group letters indicate a closer relationship than those further apart. More spatial variability in the meta-groups is obvious in the Glenelg catchment (shown in red), than that in the Hopkins (blue) and Portland (green) catchments.

The meta-group classifications support the idea that there was a difference in the hydrological systems of the three major catchments of the Glenelg Hopkins region (Figure 10). Interestingly, the amount of spatial variation did not decrease when the meta-group assignments of the original ALOC classes were examined, suggesting that hydrologic responses could be very different in areas that are quite close together. In the Glenelg River catchment (Figure 10, shown in red), there is obvious spatial variation in the assigned hydrological classes. Of particular interest are the two catchments in the north-east of the catchment that contain Rocklands Reservoir and the majority of the Grampians ranges, as they each consist of five different hydrological meta-groups (E, F, H, I & J). Even though the meta-groups represented in those particular catchments occupy a small area, they are still all present in the resampled classification (Figure 10, bottom row). While the differences were less pronounced in the Hopkins River catchment (Figure 10, outlined in blue), there was some spatial variability in classes in the north (dominated by meta-group I, with some small patches comprised of meta-group E), while most of the catchment belongs to group I and the two southernmost catchments belong to meta-group G. There was even less variation in the classification of the Portland catchment (Figure 10, outlined in green) with meta-group I dominating that catchment. Nonetheless, there were small areas of groups F and J in the south-west catchments of the catchment. Visual examination of the PCA and the resampled classifications showed little difference to that observed in the original 30-m classifications. The most obvious change was the small area in the southern catchment of the Hopkins River catchment (outlined in blue), and the easternmost parts of the Portland catchment (outlined in green), that was classified as meta-group J in the PCA classifications.

### Relationships between the regionalisation and hydrologic indices

A total of 201gauges were deemed suitable for inclusion in the analysis based on the criteria specified above. With the exception of groups A, K and H, all regions had multiple suitable gauges.

There were significant differences among groups based on their hydrologic indices for both the original (pseudo-*F*_1,7_ = 3.655, *p* = 0.001) and the bootstrapped datasets (pseudo-*F*_1,7_ = 9.304, *p* = 0.001). *A posteriori* pairwise comparisons of groups using the original dataset indicated that the differences were significant (*p*≤0.05) between all groups with the exception of pairs B:E, C:D, C:E and F:I. The pairwise comparisons on the bootstrapped dataset were all significant (*p*<0.05).

The ability of CAP to correctly classify cases within the original dataset based on their hydrologic indices was relatively poor, with only 92 samples correctly classified, but the model was statistically significant (48%, *m* = 30, *p* = 0.001). Stream gauges from meta-group C had the lowest classification accuracy with only 10% of gauges being successfully allocated. The highest classification accuracy was observed for both meta-groups B and G, with 67% of gauges correctly allocated to each. The bootstrap dataset performed better with 253 samples being correctly classified (66%, *m* = 28, *p* = 0.001). The lowest classification accuracy was observed for meta-group E with only 47% of gauges being correctly allocated. The highest classification accuracy was meta-group I with 74% of gauges correctly allocated.

When 20% of cases were used as a validation sample within the bootstrapped dataset, CAP performed reasonably, with 208 of the gauges correctly classified (67%, *m* = 32, *p* = 0.001). The lowest classification accuracy was observed for meta-group J with 58% of gauges being correctly allocated. The highest classification accuracy was meta-group F with 77% of gauges correctly allocated. Agreement between the samples that were allocated to new groups and their original group was relatively high at 60% (κ = 0.53). The lowest successful group allocation was observed for meta-group E where only 20% of gauges were correctly classified. The highest allocation accuracy was observed for meta-group D where 100% of gauges were correctly classified.

Pearson's correlation indicated there was a statistically-significant positive relationship between the number of gauges in each group and the number of gauges classified correctly from each group (Figure S9) for the original (*r* = 0.91, *p* = 0.002, *n* = 8), the bootstrapped dataset (*r* = 0.93, *p* = 0.001, *n* = 8), and the bootstrapped dataset with the validation samples (*r* = 0.96, *p*<0.001, *n* = 8). This indicated that the model was most likely to correctly classify groups that were common in the dataset, with uncommon groups being correctly classified less often. All classifications performed highly favourably compared to a random allocation of cases among 11 groups which, assuming equal sample sizes, results in 9% of cases being correctly classified.

## Discussion

By incorporating a number of environmental variables likely to influence regional hydrology and a range of non-parametric statistical and classification methods, this study aimed to generate a hydrologic landscape classification that did not require the use of an *a priori* selection of a spatial unit such as a catchment. The main objective of the approach was to see whether it was possible to create a classification that could preserve the environmental and hydrological variability that are known to influence streamflows within and between catchments that has typically been lost in previous regionalisation studies. An analysis of the ability of the classification to differentiate between streams from each group based on a number of streamflow indices was also undertaken.

### Difference to previous regionalisation studies

Inductive methods of hydrologic regionalisation have been popular in the past (see Table 2 in Olden et al. [9]) and, while there have been a number of studies that have focused on deductive methods (see Table 1 in Olden et al. [9]), the choice of variables, their resolution (temporal and spatial), the classification method, the spatial scale of the classification and the number of groups are all known to influence deductive classifications [9], [14]. While the final product of deductive methods is a spatial mosaic of independent hydrologic types, the final classifications do not always only identify hydrologic variation [10], [12]. Inter-catchment variability can limit the applicability of hydrologic regionalisations to generalise and predict catchment behaviour as a function of climatic and environmental gradients [14]. As previous studies have relied on catchments, landscape units or stream sections [8], [9], [14], [19], an issue that is more apparent in deductive regionalisation studies is the loss of small-scale spatial hydrologic variability [6], [16] as the unit of analysis gets larger. Our method relied on using an accurate supervised image classification method to extend our statistical clustering to an area covering ∼228,000 km^2^ without the need to rely on catchments, landscape units or stream sections. We opted for this approach as it is well known in the literature that there is significant flow variability within and among catchments and that the variables governing flow variability are dependent on scale [13]. While other regionalisation studies have identified that the primary drivers of catchment function are largely related to climatic gradients [14], our results suggest that a mixture of climatic, geological and environmental functions are driving catchment, and thus hydrologic, variability (Table S2, Figure S8) in our regionalisation. It would be expected, in a traditional approach to hydrologic regionalisations, that some of this variability would be lost – which could explain why other studies have largely identified climatic gradients that vary slowly with space to be the primary drivers of catchment function. Our approach allows for different classes to be represented within a single catchment, thus preserving intra-catchment variability.

### Statistical evaluation, clustering, and PCA

Traditional parametric statistical analyses and clustering algorithms such as *k*-means tend to have restrictive assumptions regarding independence of samples, multivariate normality and collinearity. The assumption of samples being distributed normally through multivariate space, for example, is unlikely to be true in most ecological and environmental datasets [25]. The approach that we employed relied on the use of non-parametric and permutation-based statistical methods in conjunction with the RF classification algorithm. This approach had far fewer assumptions relating to data normality and collinearity [27], [44], [45]. The approach also allowed the decision regarding the number of groups used in the analysis to be statistically-justified, when this decision is typically arbitrary. Our method was supported by the application of both ANOSIM and MDS to assess group separation, with each suggesting that the groups were distinct and that we had chosen an appropriate number of groups for our dataset. While not perfect (SIMPROF, by design, tests for hierarchically-related groups and we were after non-hierarchical groups), we believe this approach to be simpler, more statistically sound and more efficient than methods employed in the past which require large amount of *a posteriori* or *post-hoc* statistical testing [10], [19].

The use of PCA-transformed data was shown not to significantly affect the classification accuracy of the model, even though the PCA was only able to explain <80% of the data variability (Table 1, ALOC 23 PCA). This suggests that future classifications could be conducted on PCA-transformed datasets while still producing accurate classification schemes. Our method has essentially shown that it is possible to extract the same number of groups from PCA-transformed data as it was from the non-PCA transformed data. However, using PCA from the beginning has the potential to influence the number of groups identifiable by SIMPROF (as there are fewer data and less variance in the remaining data) and therefore influence the overall classification process. Using PCA could, however, make the process more efficient in that having a reduced number of groups to begin with could remove the need to first use non-hierarchical classification before hierarchically classifying the groups. The major benefit of using PCA-transformed data in this study was that the time to parameterise and classify the raster data with RF was substantially reduced.

### Classification by Random Forests

The non-parametric and highly accurate [27], [48] RF classifier was very successful in recovering and classifying the ALOC class information of the remaining pixels in the raster datasets. While the ALOC 23 RF models had very high classification accuracies (95%, κ = 0.94 and 92%, κ = 0.92), our hypothesis that the classification of the coarser datasets would be inferior was confirmed by the low accuracies of the ALOC 20 classifications (46%, κ = 0.42 and 47%, κ = 0.44). As our sampling density was severely limited by pixel size in the 10-km models, this further supports previous research showing that the overall and per-class accuracy of supervised classifications can be limited by pixel size [39], [40]. While the RF classifier has been shown to be robust against statistical noise and training data reduction [48], it is possible that, in this case, there were simply not enough training sites to allow for the creation of an appropriate model at the 10-km scale. This analysis used only 325 training sites (80% of *n* = 406 10-km sample points) to try to produce a classifier for 20 classes and another 33% of the training site data was excluded for OOB accuracy assessment [48]. This left the RF algorithm with only 215 points to generate the required classification trees. The two ALOC 20 100% models used 268 points (66% of *n* = 406 10-km sample points) to create an RF model but still performed poorly with OOB error rates limiting accuracies to 56% and 54%. As all classes were included in training data for the ALOC 20 100% models, their omission from the final classification (Figure 6 & 7) suggests that they, by chance, happened to be excluded from the training data selected by the bootstrapping step used to calculate OOB error. This could partly explain the high OOB error rates observed for those models. The missing classes from the ALOC 20 classifications (Figure 7, Table S3) could be similarly explained, although it is also possible that they were excluded randomly from the 80% training data at the previous step. As expected, the exclusion of data, either manually for validation purposes or automatically by RF to enable an OOB estimate, appeared to severely limit the classification accuracy when using small amounts of training data [48]. This further supports our hypothesis that limiting the study to a 10-km resolution based on a single, coarse dataset would influence the results presented here, particularly as this study was conducted over a relatively small area. If the study had been conducted at a continental scale, for example, it would be possible to generate more than 15,500 random points at a minimum distance of 10 km and, therefore, resampling the same datasets to a finer resolution (to avoid sub-sampling of pixels) would not be necessary.

The resampling of the ALOC 23 models from a 30-m to 2.5-km resolution appeared to remove some of the finer-scale spatial variability in the classifications (Figures 5 & 8). While not appearing to constitute a significant change between the original and resampled classifications (percent agreements between the resampled and original classifications were 92% (κ = 0.92) for the ALOC 23 model and 88% (κ = 0.87) for the ALOC 23 PCA model), the results of both McNemar's test and the permutation test indicated that the resampled classifications were significantly different from their equivalent 30-m classifications. This may seem like a serious drawback to the method, however, when compared to the validation dataset the accuracy of the 2.5-km ALOC 23 classification was only 4% less than that observed for the 30-m model, while the accuracy of the 2.5-km ALOC 23 PCA classification was only 5% less than that of the 30-m PCA model. While statistically the difference may be deemed significant, we contend that in reality a difference of ≤5% would likely not be ecologically or environmentally important and thus, would not affect the ability of the method to create a hydrological landscape classification that could be used to explain spatial differences in streamflow metrics. Additionally, the resampling step was performed *a posteriori* and therefore may not actually be necessary in all cases.

### Case study on spatial variability in western Victoria

The spatial variability in the classification of the Glenelg Hopkins region was most evident in the Glenelg catchment, and less so in the Hopkins and Portland catchments (Figure 9 & 10). Spatial hydrologic variability has been observed in a number of studies in the past and the strongest and most variable relationships between environmental factors and water quality and quantity have consistently been found in the Glenelg catchment. Brown et al. [17] found that the Glenelg catchment exhibited the most variability in the relationships between climate, land use and wetland extent, which may help to explain some of the variability observed here. Relationships explored in the past relating the proportion of native vegetation to in-stream salinity [35] showed strong relationships in the Glenelg and Portland catchments, but less evidence for the same relationships in the Hopkins catchment and it was suggested that this may have been due to the degraded nature of the catchment. The degraded nature of the Hopkins catchment could also explain the lack of variability observed in that catchment in this study. A spatially-varying relationship between nutrient exports and land use has also been observed in the Glenelg Hopkins region [34] although, due to the lack of suitable stream-gauge data, it is not clear whether this relationship holds for the streams and rivers of the region. However, the relationships in the region that have been described in the past [17], [34], [35] are likely to be complicated due to variations in geomorphology, groundwater levels and salt concentrations [36]; conditions that we have attempted to account for in this study. Water resource managers in the region need to take into account possible differences in intra- and inter-catchment hydrology that could drastically affect river management and restoration plans and regionalisation studies such as the one presented here could assist in identifying that variability.

### Relationships between the regionalisation and hydrologic indices

Understanding, and being able to accurately predict, streamflow characteristics in ungauged locations is crucial for ecohydrological and other studies [2], [6]. Our method set out to test a new approach to hydrological regionalisation that removed the need for catchments as a spatial unit of analysis for our statistical clustering [6], [8], [14]. However, the ability to link the results of the regionalisation to streamflow indices could have presented an issue given that we did not rely on catchments as a spatial unit. The results supported our hypothesis that our method would be able to identify and preserve inter- and intra-catchment variability. Pairwise comparisons suggested that, even in the original dataset (*n* = 201), there was enough variability in our 32 streamflow indices to separate all but four pairs (of a total of 28 pairs). A simple bootstrap (with replacement) to *n* = 383 gauges was sufficient for all groups to be easily identified as distinct suggesting there is a minimum number of gauges for the approach to be reliable (likely to relate to the number of each individual class in the dataset). In addition, CAP demonstrated that the regionalisation was able to discriminate among streams from different groups. While the classification was not perfect, the analysis conducted on the bootstrap datasets indicated that the stream gauges could be classified correctly significantly better than chance alone, and that gauges from one class could be correctly classified 100 percent of the time. In general, common classes were correctly classified more frequently than uncommon classes. While the correlation between the number of gauges in each class and the number of correctly-allocated samples was significant, there was no clear threshold (i.e. the relationship was linear), so it is difficult to identify a single minimum number of gauges that could be implemented to ensure that results met pre-defined criteria for reliability. Therefore, future users should interpret results for rare groups with caution. However, we believe that, based on this preliminary assessment of our method, the results illustrate that there is promise in the method for categorising regions, particularly in the absence of comprehensive streamflow data as is the case in many regions in Australia and elsewhere. Further investigation of the validation (i.e. linking the regionalisation to streamflow indices) using more in-depth data mining approaches (e.g. decision trees [49]) is likely to produce even greater classification success.

## Conclusions

Hydrologic classifications are increasingly being employed in the management and research of aquatic resources. Our approach differed from inductive hydrological regionalisation where membership is defined quantitatively based on metrics of stream flow, and traditional deductive regionalisation which require the use of catchments or other appropriate spatial units. Instead, membership of pixels was defined qualitatively with the random forest classifier based on a statistical classification of a number of environmental variables that we believe could have a direct influence on the hydrologic cycle. In essence, we present a method that allowed the creation of spatially-independent hydrological regions; these regions represent a series of fundamental hydrologic landscape units that exist in multiple locations depending on environmental similarity rather than a combination of environment and streamflow-metric response similarity. To our knowledge, the application of deductive reasoning and hybrid classification is a novel approach in hydrological regionalisation. This method has removed the need to rely on a spatial unit specified *a priori*, such as a catchment or ecoregion, and has allowed the preservation of intra-catchment variability. Thus, it should be useful in the spatial explanation and prediction of streamflow responses that are known, or suspected, to vary within catchments. The ability of our regionalisation to discriminate among streams from different groups based on their range of flow indices highlights the value of this approach, particularly in regions where streamflow data are lacking.

## Supporting Information

Figure S1 **MDS Analysis plots for the ALOC 23 and ALOC 20 models.** Top row shows the ALOC 23 and ALOC 20 groups, while the bottom row shows the ALOC 23 and ALOC 20 meta-group plots. Legends in the top row represent the meta-groups.
(PDF)

Figure S2 **ALOC 23 and ALOC 20 dendrogram demonstrating the hierarchical relationships between the non-hierarchical groups as defined from the ALOC group averages using SIMPROF.** Letters in green represent the meta-groups each combination of non-hierarchical groups belongs to.
(PDF)

Figure S3 **BioClim variable distributions across each of the ALOC 23 meta-groups.**
(PDF)

Figure S4 **Groundwater variable distributions across each of the ALOC 23 meta-groups.** Note that observations >>30,000 are missing from GW_TDS.
(PDF)

Figure S5 **Landscape variable distributions across each of the ALOC 23 meta-groups.**
(PDF)

Figure S6 **Soil variable distributions across each of the ALOC 23 meta-groups.**
(PDF)

Figure S7 **Climate variable distributions across each of the ALOC 23 meta-groups.**
(PDF)

Figure S8 **Variable contribution to each of the hierarchical meta-groups calculated using SIMPER on a standardised Euclidean distance matrix.** Any variables contributing <5% to the variance were pooled together and are represented by ALL_OTHER_VARS. Missing groups contained only one ALOC cluster and therefore % variable contribution could not be calculated with SIMPER.
(PDF)

Figure S9 **The correlation between the number of samples from each class and the number of samples correctly classified by CAP was highly significant, with the linear relationship between the variables for each sample illustrated in the figure.** However, as the relationship was linear there was no clear threshold suggesting a minimum number of gauges needed to guarantee an acceptable level of accuracy. The CAP on the original dataset (*n* = 201) was quite poor (classification accuracy  = 48%, *m* = 30, *p* = 0.001), while the bootstrapped dataset (*n* = 383) was a significant improvement (classification accuracy  = 66%, *m* = 28, *p* = 0.001). CAP on the bootstrap dataset with a 20% validation sample also performed reasonably (classification accuracy  = 67%, *m* = 32, *p* = 0.001).
(PDF)

Table S1 **The variables used in the creation of the hydrological regionalisation.** A number of variables describing the storage, transport and release of surface water, groundwater and atmospheric water were included in the analysis. * The DTM data was resampled to 30 m to enable geo-TIFF compatibility with ENVI 4.8.
(PDF)

Table S2 **SIMPER results from PRIMER 6. Numbers represent % contribution of each of the variables to the ALOC 23 and ALOC 20 meta-groups on a standardised Euclidean distance matrix.** Blank columns are meta-groups that only contained 1 ALOC cluster and therefore % contribution could not be calculated using SIMPER. KW  =  Kruskal-Wallis statistic, with higher values indicating a better ability of that variable to discriminate between clusters. All KW values were significant at *p*<<0.001. See Figure S9 for a graphical representation of this table.
(PDF)

Table S3 **Total accuracies and kappa statistics for the 4 RF classified models, and producer and user accuracies for each of the classes defined by the ALOC algorithm as classified by RF.** N/A indicates groups that were missing from the classified dataset as a result of exclusion from the samples used to train the RF model. In some cases, groups were absent from the 80% training data, while others were excluded by the bootstrap aggregation step used to train the RF models.
(PDF)

Code S1 **S_perm_test.R: R code for running the permutation test for testing for differences between the resampled and original classifications.**
(R) Click here for additional data file.

Data S1 **S_Kappa_ALOC23.csv: CSV file with 4 columns.** The first column is the point ID from the random subsample used for comparisons between the original and resampled classifications, the remaining columns are the “true” class from the ALOC clustering, the class extracted from the location of the 30-m RF classification, and the class extracted from the 2.5-km resampled RF classification.
(CSV)
