# Supplementary material for: Hydrologic Landscape Regionalisation Using Deductive Classification and Random Forests
Source: PLoS One. 2014 Nov 14;9(11):e112856. doi: 10.1371/journal.pone.0112856 (PMC4232575; doi:10.1371/journal.pone.0112856)
Supplement: Figure S1 — MDS Analysis plots for the ALOC 23 and ALOC 20 models. Top row shows the ALOC 23 and ALOC 20 groups, while the bottom row shows the ALOC 23 and ALOC 20 meta-group plots. Legends in the top row represent the meta-groups. (PDF) [file pone.0112856.s001.pdf]

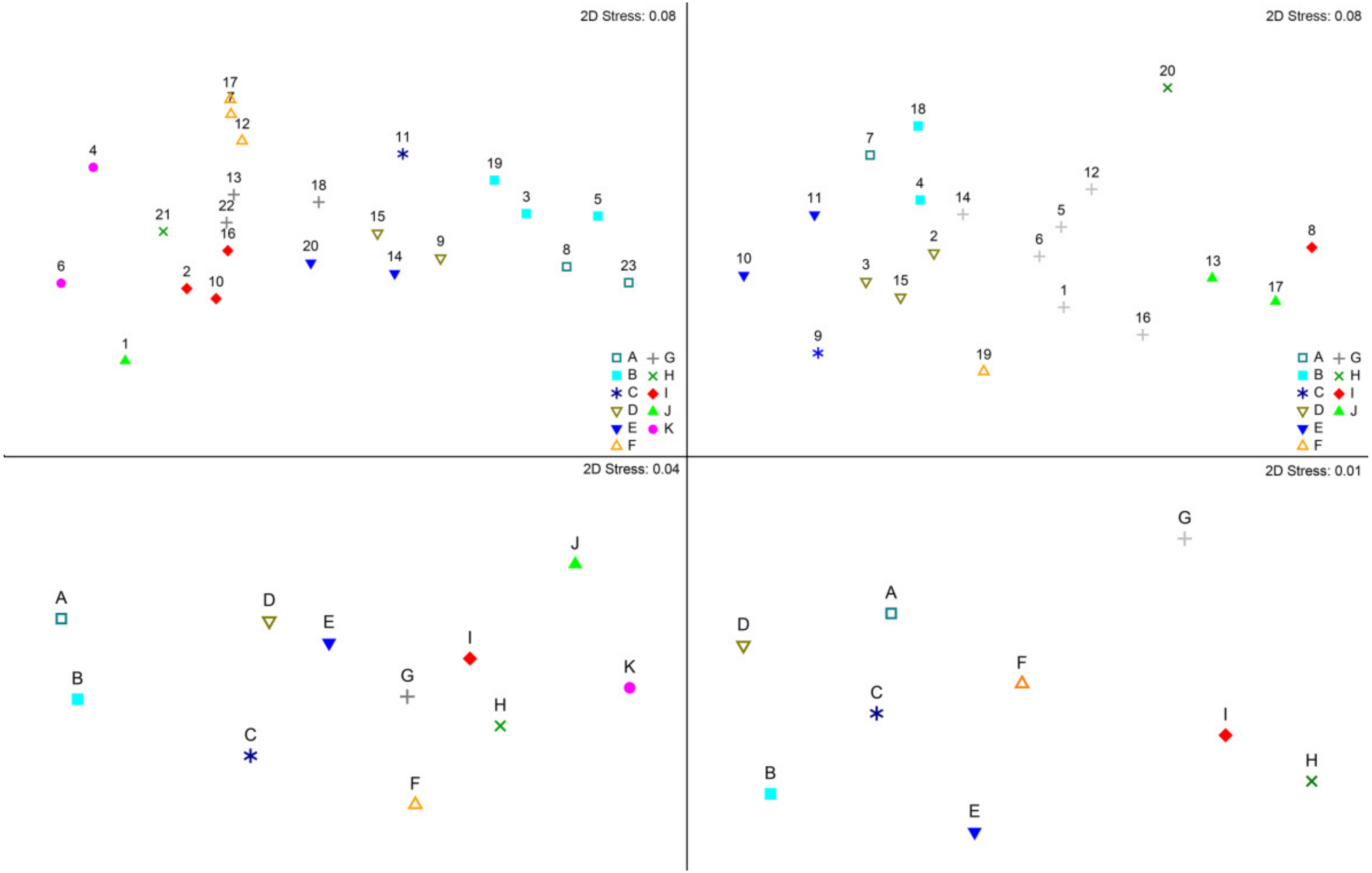

Figure S1: MDS Analysis plots for the ALOC 23 and ALOC 20 models. Top row shows the ALOC 23 and ALOC 20 groups, while the bottom row shows the ALOC 23 and ALOC 20 meta-group plots. Legends in the top row represent the meta-groups.
