# Supplementary material for: Hydrologic Landscape Regionalisation Using Deductive Classification and Random Forests
Source: PLoS One. 2014 Nov 14;9(11):e112856. doi: 10.1371/journal.pone.0112856 (PMC4232575; doi:10.1371/journal.pone.0112856)
Supplement: Figure S2 — ALOC 23 and ALOC 20 dendrogram demonstrating the hierarchical relationships between the non-hierarchical groups as defined from the ALOC group averages using SIMPROF. Letters in green represent the meta-groups each combination of non-hierarchical groups belongs to. (PDF) [file pone.0112856.s002.pdf]

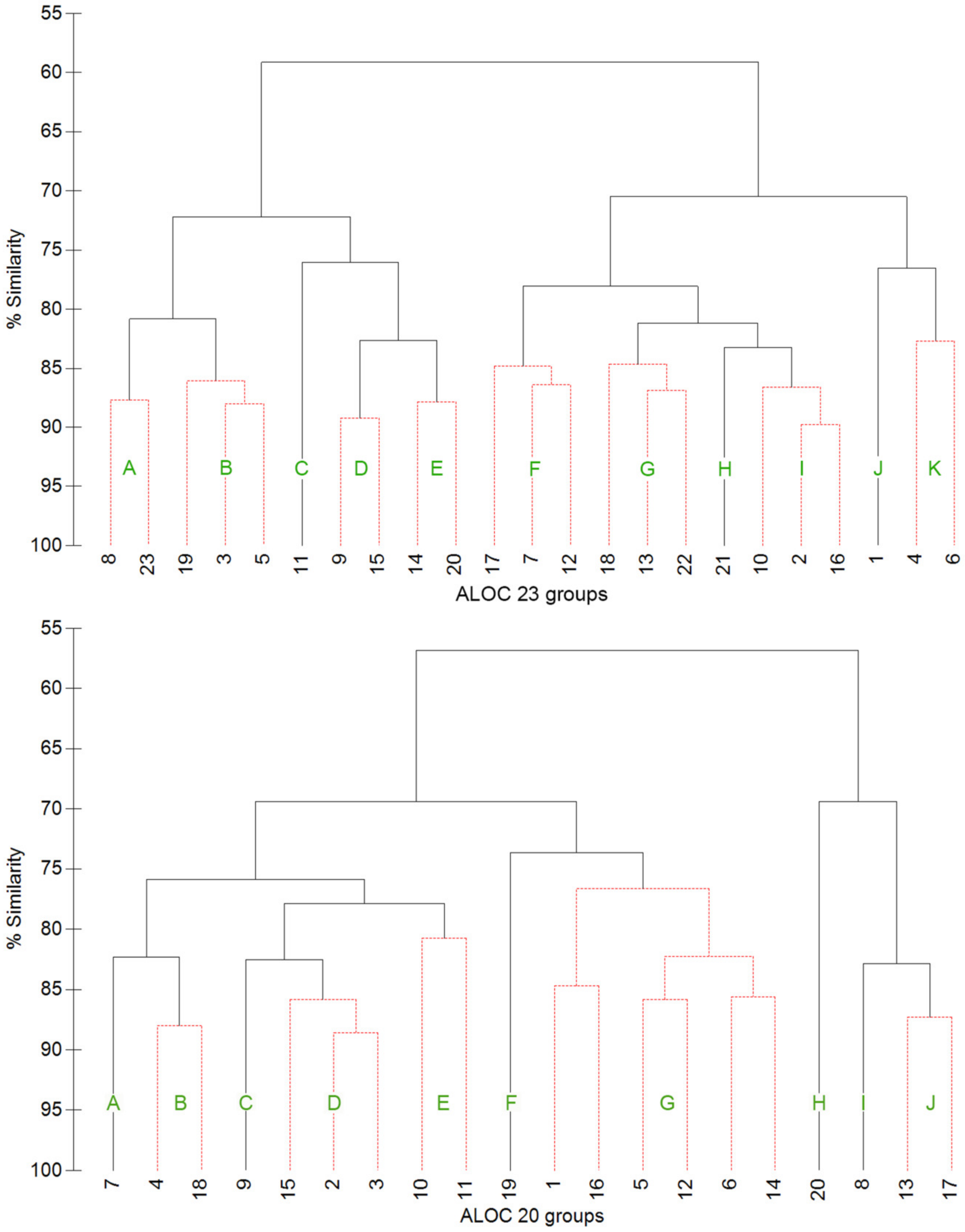

Figure S2: ALOC 23 and ALOC 20 dendrogram demonstrating the hierarchical relationships between the non-hierarchical groups as defined from the ALOC group averages using SIMPROF. Letters in green represent the meta-groups each combination of non-hierarchical groups belongs to.
