# Supplementary figures and images for: Hydrologic Landscape Regionalisation Using Deductive Classification and Random Forests
Source: PLoS One. 2014 Nov 14;9(11):e112856. doi: 10.1371/journal.pone.0112856 (PMC4232575; doi:10.1371/journal.pone.0112856)

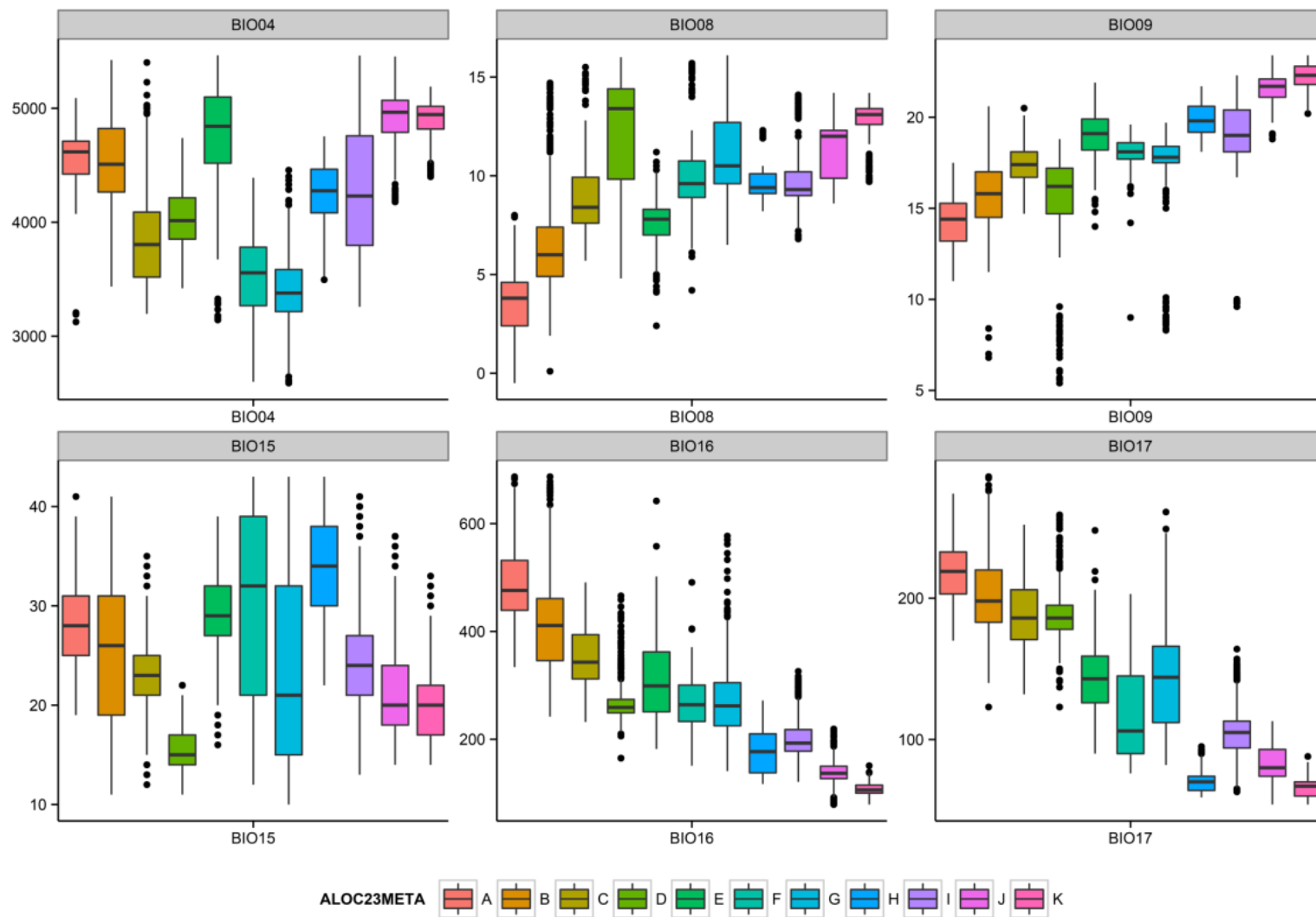

Figure S3: BioClim variable distributions across each of the ALOC 23 meta-groups.

Supplement: Figure S3 — BioClim variable distributions across each of the ALOC 23 meta-groups. (PDF) [file pone.0112856.s003.pdf]

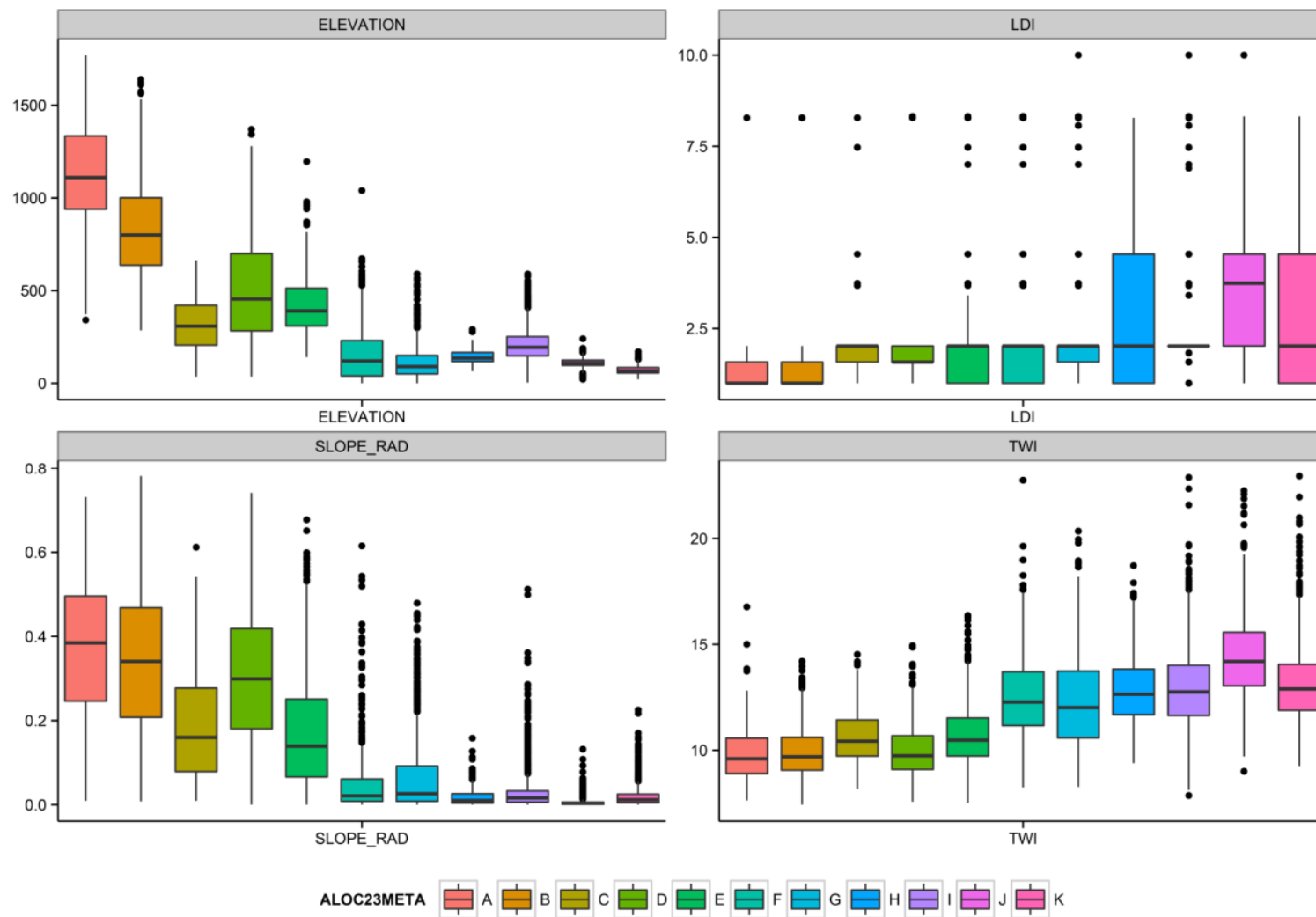

Figure S5: Landscape variable distributions across each of the ALOC 23 meta-groups.

Supplement: Figure S5 — Landscape variable distributions across each of the ALOC 23 meta-groups. (PDF) [file pone.0112856.s005.pdf]

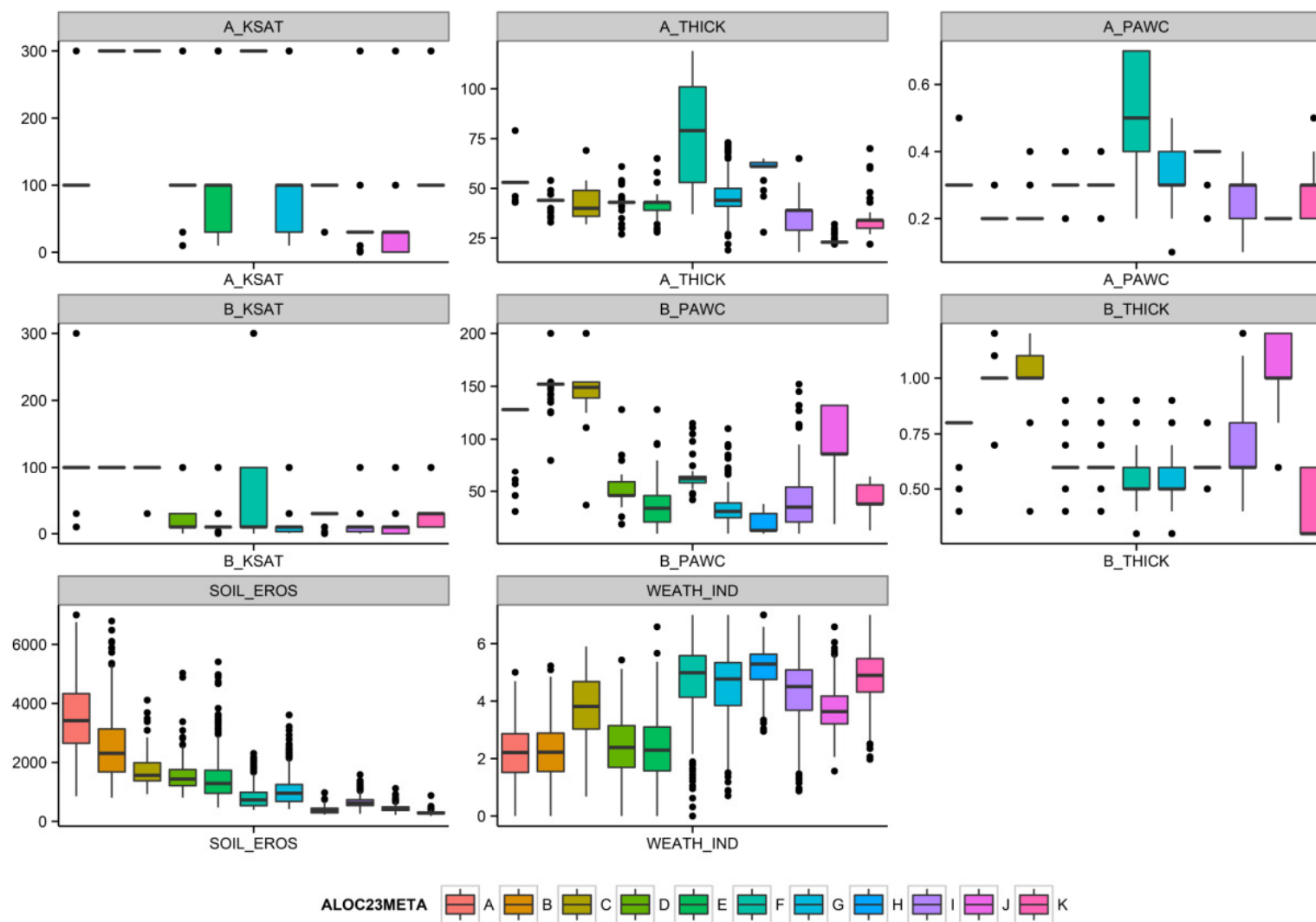

Figure S6: Soil variable distributions across each of the ALOC 23 meta-groups.

Supplement: Figure S6 — Soil variable distributions across each of the ALOC 23 meta-groups. (PDF) [file pone.0112856.s006.pdf]

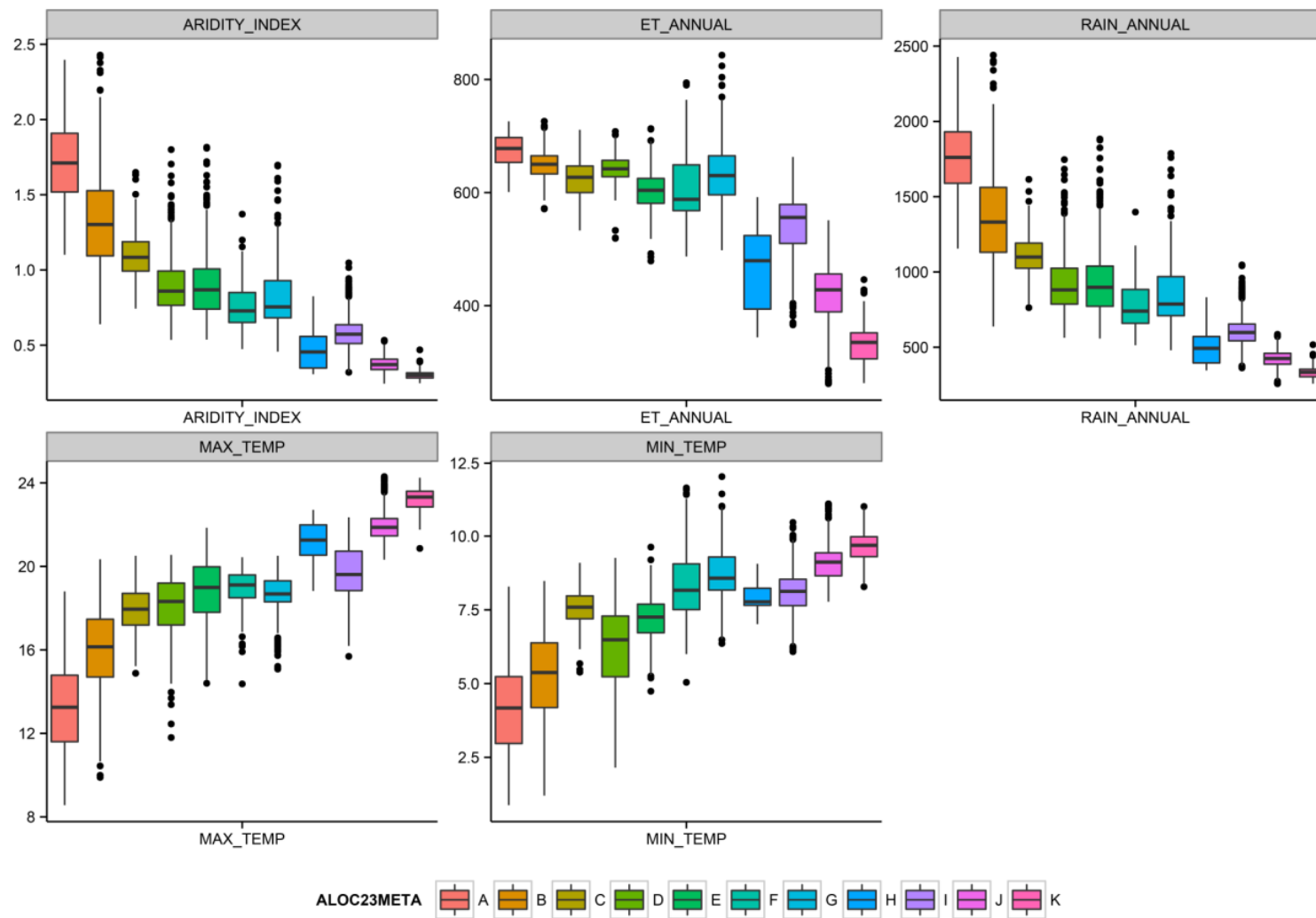

Figure S7: Climate variable distributions across each of the ALOC 23 meta-groups.

Supplement: Figure S7 — Climate variable distributions across each of the ALOC 23 meta-groups. (PDF) [file pone.0112856.s007.pdf]
