# Supplementary material for: Hydrologic Landscape Regionalisation Using Deductive Classification and Random Forests
Source: PLoS One. 2014 Nov 14;9(11):e112856. doi: 10.1371/journal.pone.0112856 (PMC4232575; doi:10.1371/journal.pone.0112856)
Supplement: Figure S4 — Groundwater variable distributions across each of the ALOC 23 meta-groups. Note that observations >>30,000 are missing from GW_TDS. (PDF) [file pone.0112856.s004.pdf]

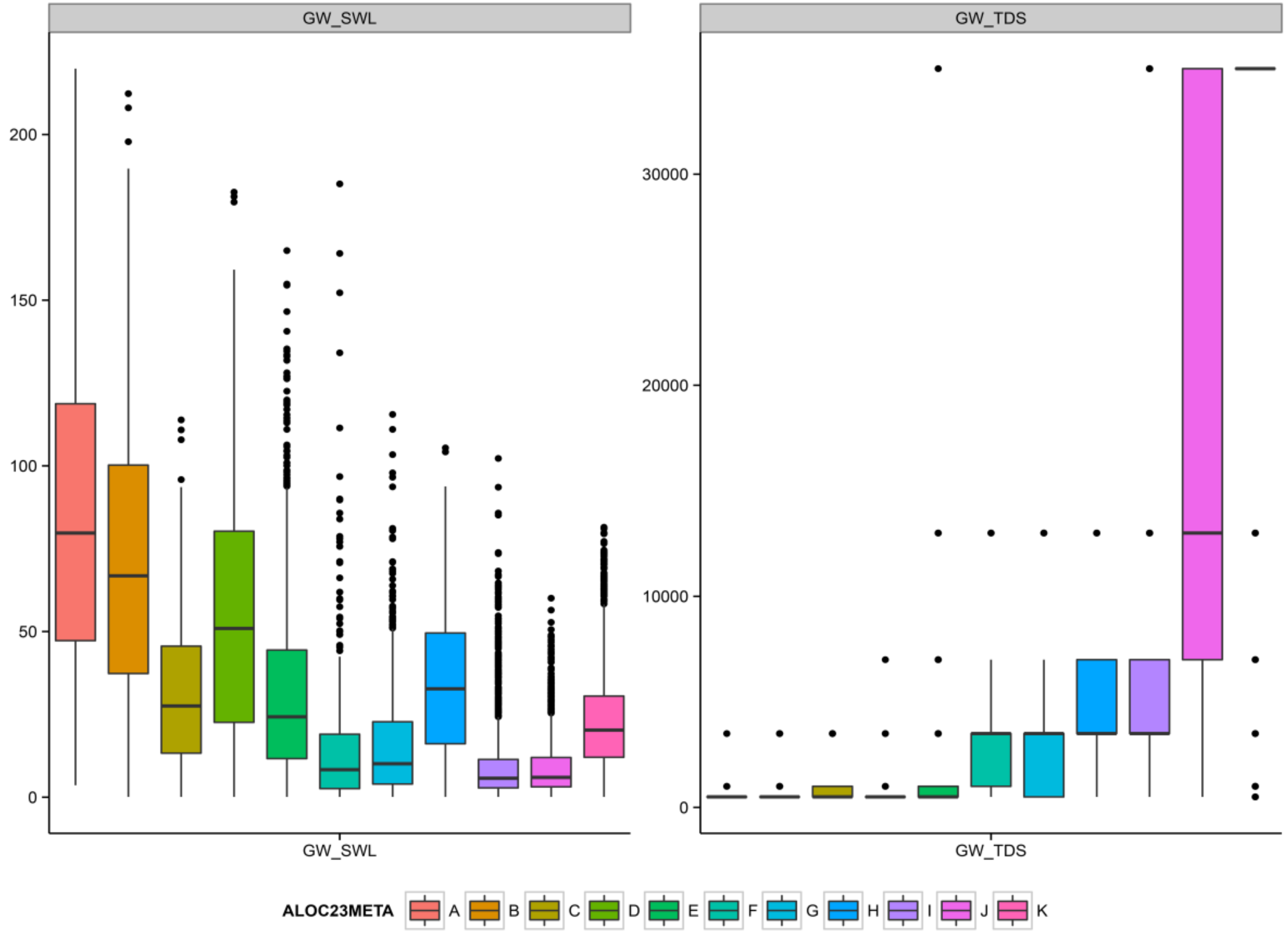

Figure S4: Groundwater variable distributions across each of the ALOC 23 meta-groups. Note that observations >> 30,000 are missing from GW_TDS.
