# Supplementary material for: Hydrologic Landscape Regionalisation Using Deductive Classification and Random Forests
Source: PLoS One. 2014 Nov 14;9(11):e112856. doi: 10.1371/journal.pone.0112856 (PMC4232575; doi:10.1371/journal.pone.0112856)
Supplement: Figure S8 — Variable contribution to each of the hierarchical meta-groups calculated using SIMPER on a standardised Euclidean distance matrix. Any variables contributing <5% to the variance were pooled together and are represented by ALL_OTHER_VARS. Missing groups contained only one ALOC cluster and therefore % variable contribution could not be calculated with SIMPER. (PDF) [file pone.0112856.s008.pdf]

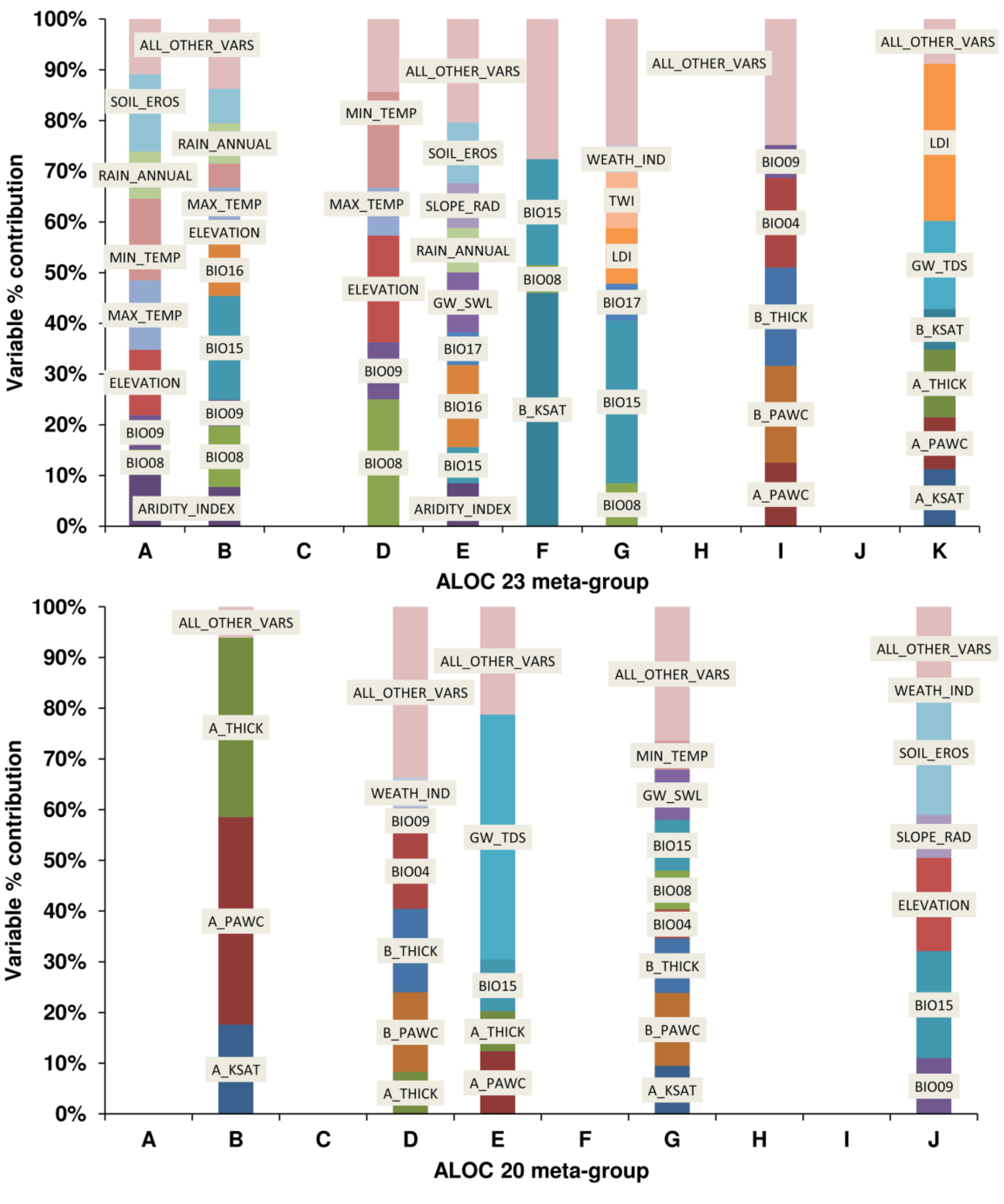

Figure S8: Variable contribution to each of the hierarchical meta-groups calculated using SIMPER on a standardised Euclidean distance matrix. Any variables contributing <5% to the variance were pooled together and are represented by ALL_OTHER_VARS. Missing groups contained only one ALOC cluster and therefore % variable contribution could not be calculated with SIMPER.
