# Supplementary material for: Hydrologic Landscape Regionalisation Using Deductive Classification and Random Forests
Source: PLoS One. 2014 Nov 14;9(11):e112856. doi: 10.1371/journal.pone.0112856 (PMC4232575; doi:10.1371/journal.pone.0112856)
Supplement: Figure S9 — The correlation between the number of samples from each class and the number of samples correctly classified by CAP was highly significant, with the linear relationship between the variables for each sample illustrated in the figure. However, as the relationship was linear there was no clear threshold suggesting a minimum number of gauges needed to guarantee an acceptable level of accuracy. The CAP on the original dataset (n = 201) was quite poor (classification accuracy = 48%, m = 30, p = 0.001), while the bootstrapped dataset (n = 383) was a significant improvement (classification accuracy = 66%, m = 28, p = 0.001). CAP on the bootstrap dataset with a 20% validation sample also performed reasonably (classification accuracy = 67%, m = 32, p = 0.001). (PDF) [file pone.0112856.s009.pdf]

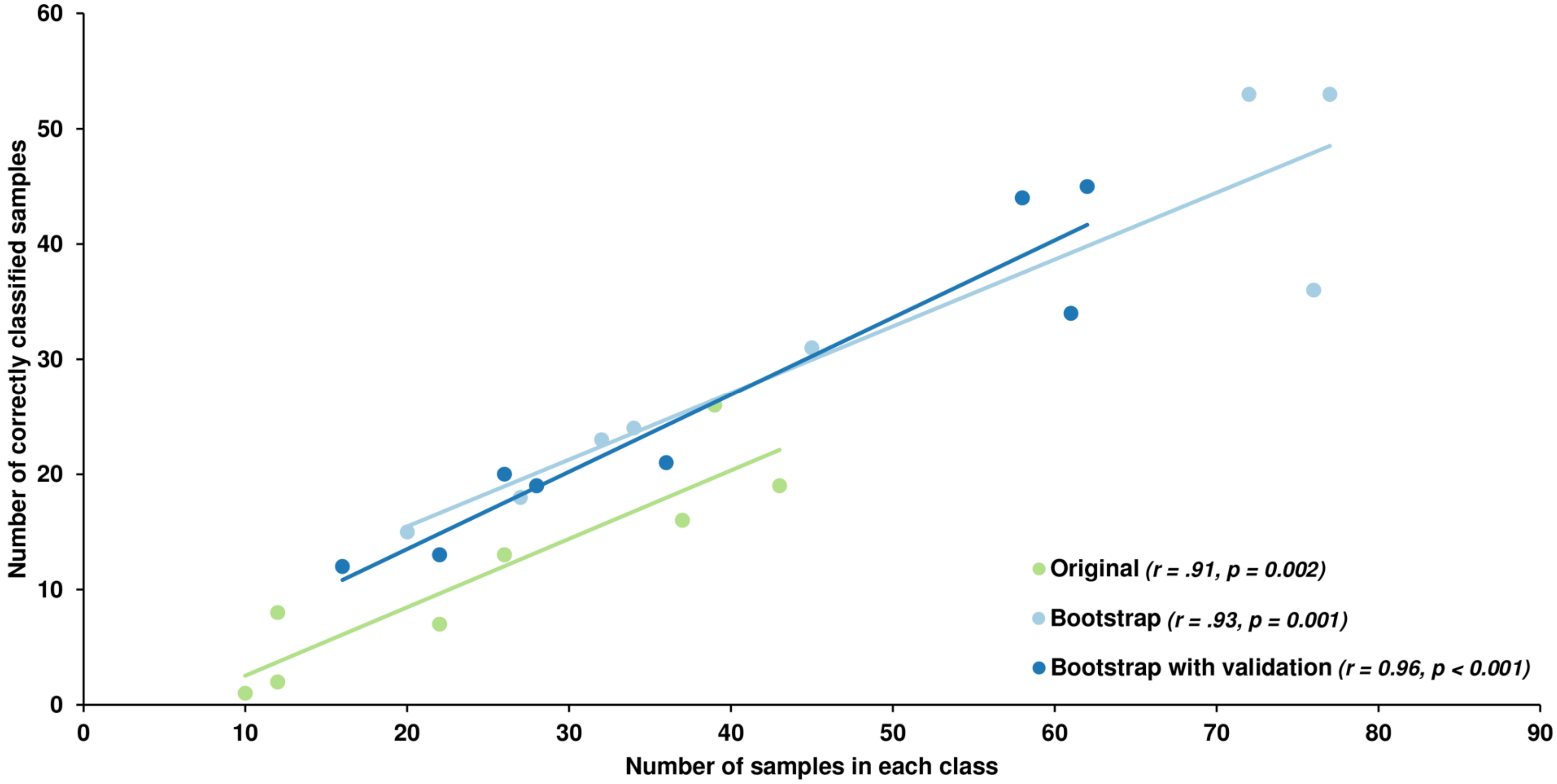

Figure S9: The correlation between the number of samples from each class and the number of samples correctly classified by CAP was highly significant, with the linear relationship between the variables for each sample illustrated in the figure. However, as the relationship was linear there was no clear threshold suggesting a minimum number of gauges needed to guarantee an acceptable level of accuracy. The CAP on the original dataset (n = 201) was quite poor (classification accuracy = 48%, m = 30, p = 0.001), while the bootstrapped dataset (n = 383) was a significant improvement (classification accuracy = 66%, m = 28, p = 0.001). CAP on the bootstrap dataset with a 20% validation sample also performed reasonably (classification accuracy = 67%, m = 32, p = 0.001).
