# Supplementary material for: Hydrologic Landscape Regionalisation Using Deductive Classification and Random Forests
Source: PLoS One. 2014 Nov 14;9(11):e112856. doi: 10.1371/journal.pone.0112856 (PMC4232575; doi:10.1371/journal.pone.0112856)
Supplement: Table S1 — The variables used in the creation of the hydrological regionalisation. A number of variables describing the storage, transport and release of surface water, groundwater and atmospheric water were included in the analysis. * The DTM data was resampled to 30 m to enable geo-TIFF compatibility with ENVI 4.8. (PDF) [file pone.0112856.s010.pdf]

Table S1: The variables used in the creation of the hydrological regionalisation. A number of variables describing the storage, transport and release of surface water, groundwater and atmospheric water were included in the analysis. * The DTM data was resampled to 30 m to enable geo-TIFF compatibility with ENVI 4.8.

Variable Name	*Abbreviation*	*Description*	*Source*	*Resolution*
Plant AWC – A Horizon	A_PAWC	Plant available water capacity A horizon (mm)	[1]	1 km
Plant AWC – B Horizon	B_PAWC	Plant available water capacity B horizon (mm)	[1]	1 km
Layer Thickness – A Horizon	A_THICK	The weighted average A horizon thickness (m)	[1]	1 km
Layer Thickness – B Horizon	B_THICK	The weighted average B horizon thickness (m)	[1]	1 km
Saturated Conductivity – A Horizon	A_KSAT	The weighted average of median A horizon saturated hydraulic conductivity (mm/hour)	[1]	1 km
Saturated Conductivity – B Horizon	B_KSAT	The weighted average of median B horizon saturated hydraulic conductivity (mm/hour)	[1]	1 km
Soil Erosivity Index (R-Factor)	SOIL_EROS	Rainfall erosivity (MJ.mm/ha.hour.year) for soils.	[2]	5 km
Groundwater Static Water Level	GW_SWL	Static water level of groundwater aquifer (MASL)	[3]	100 m
Groundwater Total Dissolved Solids	GW_TDS	Groundwater aquifer salinity (TDS)	[4]	100 m
Mean Maximum Temperature	MAX_TEMP	Mean maximum annual temperature (°C)	[5]	2.5 km
Mean Minimum Temperature	MIN_TEMP	Mean minimum annual temperature (°C)	[5]	2.5 km
BIOCLIM 4	BIO04	Temperature Seasonality	[6]	1 km
BIOCLIM 8	BIO08	Mean Temperature Of Wettest Quarter	[6]	1 km
BIOCLIM 9	BIO09	Mean Temperature Of Driest Quarter	[6]	1 km
Mean Annual Rainfall	RAIN_ANNUAL	Mean annual rainfall (mm)	[5]	2.5 km
BIOCLIM 15	BIO15	Precipitation Seasonality	[6]	1 km
BIOCLIM 16	BIO16	Precipitation Of Wettest Quarter	[6]	1 km
BIOCLIM 17	BIO17	Precipitation Of Driest Quarter	[6]	1 km
Mean Annual Evapotranspiration	ET_ANNUAL	Mean annual evapotranspiration (mm)	[5]	10 km
Aridity Index	ARIDITY_INDEX	Mean annual precipitation / Potential evapotranspiration (mm/mm)	Produced in ArcGIS 10.1	2.5 km/10 km
Landscape Development Intensity	LDI	An index for measuring anthropogenic loadings on landscapes. Values range from 1-10 on a normalised natural log scale, with higher values indicating more intensive landscape development/use.	Produced in ArcGIS 10.1 [7,8]	50 m
Elevation	ELEV	Elevation (metres above mean sea level [MASL])	[9]	30 m*
Slope	SLOPE_RAD	Slope steepness from elevation dataset (radians)	Produced in ArcGIS 10.1	30 m
Topographic Wetness Index	TWI	High TWI values represent drainage depressions; lower values represent crests and ridges. TWI = $\ln(a/\tan B)$, where a = upstream contributing area, B = slope in radians	Produced in ArcGIS 10.1	30 m
Weathering Intensity Index	WEATH_IND	Ranges in value from 1 – 6. Low values indicate unweathered bedrock; high values indicate heavily weathered rock.	[10]	100 m

References

1. Western AW, McKenzie N (2006) Soil Hydrologic Properties of Australia. Australia: CRC for Catchment Hydrology.

2. Lu H, Yu B (2002) Spatial and seasonal distribution of rainfall erosivity in Australia. Soil Research 40: 887-901.

3. Department of Sustainability and Environment (2012) Victorian Statewide Watertable Mapping. Melbourne: Department of Sustainability and Environment.

4. Department of Sustainability and Environment (2012) Victorian Statewide Watertable Salinity Mapping. Melbourne: Department of Sustainability and Environment.

5. Bureau of Meteorology (2013) Bureau of Meteorology gridded climate data, available from: http://www.bom.gov.au/climate/averages/climatology/gridded-data-info/gridded-climate-data.shtml

6. Hijmans RJ, Cameron SE, Parra JL, Jones PG, Jarvis A (2005) Very high resolution interpolated climate surfaces for global land areas. International Journal of Climatology 25: 1965-1978.

7. Brown MT, Vivas MB (2005) Landscape development intensity index. Environmental Monitoring and Assessment 101: 289-309.

8. Bureau of Rural Sciences (2010) Catchment Scale Land Use Mapping for Australia Update March 2010. Canberra: Bureau of Rural Sciences.

9. Department of Sustainability and Environment (2008) Vicmap Elevation, DTM 20m. Melbourne: Department of Sustainability and Environment.

10. Wilford J (2012) A weathering intensity index for the Australian continent using airborne gamma-ray spectrometry and digital terrain analysis. Geoderma 183-184: 124-142.
