# Supplementary material for: Hydrologic Landscape Regionalisation Using Deductive Classification and Random Forests
Source: PLoS One. 2014 Nov 14;9(11):e112856. doi: 10.1371/journal.pone.0112856 (PMC4232575; doi:10.1371/journal.pone.0112856)
Supplement: Table S2 — SIMPER results from PRIMER 6. Numbers represent % contribution of each of the variables to the ALOC 23 and ALOC 20 meta-groups on a standardised Euclidean distance matrix. Blank columns are meta-groups that only contained 1 ALOC cluster and therefore % contribution could not be calculated using SIMPER. KW = Kruskal-Wallis statistic, with higher values indicating a better ability of that variable to discriminate between clusters. All KW values were significant at p<<0.001. See Figure S9 for a graphical representation of this table. (PDF) [file pone.0112856.s011.pdf]

Table S2: SIMPER results from PRIMER 6. Numbers represent % contribution of each of the variables to the ALOC 23 and ALOC 20 meta-groups on a standardised Euclidean distance matrix. Blank columns are meta-groups that only contained 1 ALOC cluster and therefore % contribution could not be calculated using SIMPER. KW = Kruskal-Wallis statistic, with higher values indicating a better ability of that variable to discriminate between clusters. All KW values were significant at $p << 0.001$. See Supporting Figure 9 for a graphical representation of this table.

		ALOC 23 meta-group												*ALOC 20 meta-group*									
		A	B	C	D	E	F	G	H	I	J	K		A	B	C	D	E	F	G	H	I	J
Original groups		8,23	3,5,19	11	9,15	14,20	7,12,17	13,18,22	21	2,10,16	1	4,6		7	4,18	9	2,3,15	10,11	19	1,5,6,12,14,16	20	8	13,17
Avg. Squared Dist .		4.70	6.03		4.18	3.19	9.58	6.19		5.36		11.77			9.07		4.44	13.45		10.61			5.8
Variables	*KW*												*KW*										
A_KSAT	3885.0	0	0		0	1.0	0	0.1		0		11.2	227.1		17.6		1.5	2.2		9.5			0
A_PAWC	2770.2	0	0.1		0	0	4.8	0.1		12.6		10.2	198.7		40.9		3.9	12.4		0.3			0
A_THICK	3269.4	0	0		0	0.1	4.5	0.4		4.8		13.3	157.0		35.4		8.3	7.9		1.4			0
ARIDITY_INDEX	4486.7	10.1	8.3		3.0	8.5	0.3	3.0		0.4		0	351.9		0		1	0.1		1.6			4.3
B_KSAT	2746.8	0.1	0		0.1	0	46.0	0.5		0		8.0	176.2		1.8		0.1	0.9		3.6			0
B_PAWC	3277.6	0.3	0.1		0.4	1.3	0.7	0.2		19.1		0	152.3		2		15.7	0.7		14.4			0.1
B_THICK	3639.9	0.1	0		0.2	0.1	1.1	0.5		19.5		2.8	226.5		0		16.5	2.8		10.8			0
BIO04	3235.3	0.7	3.2		4.4	0.1	2.8	1.9		17.7		0.6	209.6		0.1		14.9	2.1		5.7			0.1
BIO08	3268.8	5.0	12.8		25.0	3.2	5.5	8.5		0.4		0.3	169.7		0.5		1.9	2.4		7.7			0.3
BIO09	3907.6	6.8	5.6		11.3	2.7	0	0.1		6.5		0.1	301.1		0.1		5	1.2		1.6			11
BIO15	1713.7	3.6	21.9		0.1	7.1	20.9	32.0		3.7		1.9	100.5		0.1		3.3	10.2		10			21.1
BIO16	4383.7	0.3	10.9		0.7	16.2	0.7	3.5		0.7		0	335.3		0		1.3	0.3		2.8			4.3
BIO17	4226.6	0.7	2.3		0.6	6.5	3.0	7.3		0.4		0	344.4		0.1		0.9	0		0.9			1.5
ELEVATION	3687.4	12.9	5.1		21.0	3.5	1.0	0.2		0.6		0	192.5		0.1		1.9	0.1		2.5			18.4
ET_ANNUAL	4204.7	0.1	0.1		0	0.5	2.1	2.0		2.2		0	318.3		0.2		3.6	1.8		0.3			0.1
GW_SWL	1933.6	0	0.8		1.1	11.8	0.1	1.5		0.2		2.1	151.6		0.1		0.1	1.9		9.9			1.7
GW_TDS	3644.2	0	0		0	0.3	0.1	0.1		0.1		17.3	260.7		0		0.9	48.4		0			0
LDI	1240.2	1.8	0		0.4	1.1	2.1	10.9		1.6		30.9	86.0		0.3		0.5	2		0.8			0
MAX_TEMP	4014.4	13.7	6.9		9.5	2.1	0.1	0.6		3.2		0	306.2		0		0.9	0.1		1.5			2.5
MIN_TEMP	3640.2	16.1	5.0		18.8	2.0	2.8	0		0.9		0.2	206.0		0		0.2	0.1		5.7			0.1
RAIN_ANNUAL	4486.2	9.3	8.5		2.3	8.8	0.5	3.4		0.3		0	349.9		0		0.5	0		0.7			1.2
SLOPE_RAD	2870.5	1.6	0.6		0.3	8.8	0.1	3.0		0		0	215.2		0.1		3.9	0.6		0.4			8.5
SOIL_EROS	4252.9	15.2	7.3		0.4	12.0	0.5	3.8		0		0	342.7		0.1		2.5	1.6		2.2			24.6
TWI	1990.9	1.5	0.2		0.4	2.3	0.2	11.0		0.7		0.5	148.8		0		4.8	0.4		1.7			0.2
WEATH_IND	2459.4	0.1	0.4		0	0.1	0.1	5.3		4.7		0.3	174.5		0.5		6	0		4.2			0
