# Supplementary material for: Hydrologic Landscape Regionalisation Using Deductive Classification and Random Forests
Source: PLoS One. 2014 Nov 14;9(11):e112856. doi: 10.1371/journal.pone.0112856 (PMC4232575; doi:10.1371/journal.pone.0112856)
Supplement: Table S3 — Total accuracies and kappa statistics for the 4 RF classified models, and producer and user accuracies for each of the classes defined by the ALOC algorithm as classified by RF. N/A indicates groups that were missing from the classified dataset as a result of exclusion from the samples used to train the RF model. In some cases, groups were absent from the 80% training data, while others were excluded by the bootstrap aggregation step used to train the RF models. (PDF) [file pone.0112856.s012.pdf]

Table S3: Total accuracies and kappa statistics for the 4 RF classified models, and producer and user accuracies for each of the classes defined by the ALOC algorithm as classified by RF. N/A indicates groups that were missing from the classified dataset as a result of exclusion from the samples used to train the RF model. In some cases, groups were absent from the 80% training data, while others were excluded by the bootstrap aggregation step used to train the RF models.

Model	ALOC 23 (94.9%, $\kappa = 0.94$)		ALOC 23 PCA (92.1%, $\kappa = 0.92$)		ALOC 20 (46.1%, $\kappa = 0.42$)		ALOC 20 PCA (47.4%, $\kappa = 0.44$)	
ALOC Class	Producer	User	Producer	User	Producer	User	Producer	User
ALOC 01	99.3	95.2	99.3	91.2	0.0	0.0	0.0	0.0
ALOC 02	96.2	92.1	93.6	92.5	40.0	40.0	40.0	66.7
ALOC 03	96.6	83.6	89.7	83.9	80.0	80.0	90.0	90.0
ALOC 04	95.9	98.6	95.9	100	66.7	66.7	66.7	80.0
ALOC 05	92.7	92.7	90.9	90.9	20.0	100	20.0	100
ALOC 06	99.2	99.6	99.6	100	0.0	0.0	0.0	0.0
ALOC 07	100	100	91.1	100	N/A	N/A	N/A	N/A
ALOC 08	88.2	93.8	58.8	83.3	0.0	0.0	0.0	0.0
ALOC 09	83.3	96.8	72.2	89.7	71.4	83.3	71.4	83.3
ALOC 10	94.1	100	97.1	93.0	88.9	88.9	88.9	100
ALOC 11	91.7	94.8	90.0	88.5	33.3	66.7	33.3	100
ALOC 12	100	96.1	100	94.2	N/A	N/A	N/A	N/A
ALOC 13	96.9	98.9	90.6	93.6	60.0	75.0	40.0	100
ALOC 14	81.1	89.6	91.9	86.1	0.0	0.0	16.7	25.0
ALOC 15	93.7	92.2	90.5	85.1	0.0	0.0	14.3	33.3
ALOC 16	91.0	93.6	87.6	90.1	100	33.3	0.0	0.0
ALOC 17	100	100	100	100	100	100	100	100
ALOC 18	95.2	95.2	85.7	88.5	0.0	0.0	0.0	0.0
ALOC 19	85.3	96.7	73.5	92.6	N/A	N/A	N/A	N/A
ALOC 20	87.5	89.5	81.6	89.5	N/A	N/A	N/A	N/A
ALOC 21	98.8	96.6	95.4	95.4				
ALOC 22	96.3	94.1	89.0	90.1				
ALOC 23	100	96.0	91.7	81.5				
